# First report on atrazine monitoring in drinking water from Ijebu-North, South-West Nigeria: Human health risk evaluation and reproductive toxicity studies

**DOI:** 10.3389/ftox.2022.975636

**Published:** 2022-09-26

**Authors:** Folarin Owagboriaye, Rasheed Oladunjoye, Oladunni Adekunle, Mistura Adeleke, Titilola Salisu, Adedamola Adenekan, Abibat Sulaimon, Gabriel Dedeke, Olusegun Lawal

**Affiliations:** ^1^ Department of Zoology and Environmental Biology, Faculty of Science, Olabisi Onabanjo University Ago- Iwoye, Ago Iwoye, Ogun State, Nigeria; ^2^ Department of Environmental Management and Toxicology, College of Environmental Management, Federal University of Agriculture, Abeokuta, Ogun State, Nigeria; ^3^ Department of Pure and Applied Zoology, College of Bioscience, Federal University of Agriculture, Abeokuta, Ogun State, Nigeria

**Keywords:** environmental pollution, herbicide, health risk, atrazine monitoring, reproductive dysfunction

## Abstract

There are no available data on the level of atrazine in drinking water from rural agricultural areas in Nigeria and its potential health implications. Here, we measured atrazine residue in 69 hand-dug wells (HDW), 40 boreholes (BH), and four major streams from the six communities (Ago-Iwoye, Ijebu-Igbo, Oru, Awa, Ilaporu, and Mamu) in Ijebu North Local Government Area, Southwest Nigeria. Values of atrazine obtained were further used for the evaluation of non-carcinogenic risk associated with ingestion and dermal contact in adults and children as well as reproductive toxicity evaluation. A total of 41 HDW, 22 BH, and the four streams showed varying concentrations of atrazine, which was higher in HDW than BH and stream. Ago-Iwoye recorded the highest concentration of 0.08 mg/L in its HDW while the lowest concentration of 0.01 mg/L was recorded in HDW from Oru. Although the Hazard Index (HI) values associated with ingestion and dermal contact for children were higher than in adults, the values were below the acceptable limit for all the communities. Significant (*p* < 0.05) alterations in the oxidative stress parameters, reproductive hormones, sperm parameters, and mild testicular lesions were only observed in rats exposed to atrazine at 0.08 mg/L compared to control. But atrazine at 0.01, 0.03, and 0.04 mg/L triggered a defence mechanism capable of protecting the structural integrity of the testes and preventing reproductive dysfunction.

## Introduction

Atrazine (2-chloro-4-ethylamino-6-isopropylamino-1,3,5-triazine) is one of the major herbicides used to control pre-and post-emergence broadleaf weeds on maize farms in Nigeria (Farmsquare, 2020). Since Nigeria is one of the largest producers of maize in Africa (Adeite, 2021), farmers solely depend on atrazine to maintain or improve the rate at which maize is produced. The increasing overdependence and use of atrazine on maize farms in Nigeria is now a major public health concern of national interest.

Since contaminated drinking water is the main route of exposure to atrazine in adults and children (US EPA, 2007), the Food Quality Protection Act of 1996 (FQPA) mandated the USEPA and other bodies to monitor dietary risk associated with exposure to atrazine from community water systems (CWS). This prompted the regulatory body to establish a contamination limit of 3 μg/L for atrazine in water bodies under the safe drinking water act (US EPA, 2003).

A rural hand-dug well surveyed in Minnesota by the Minnesota Department of Agriculture (MDA) for atrazine recorded the highest atrazine concentration of 3.4 μg/L (MDH. Human Health, 2009). In addition, a national survey of herbicides in domestic well water carried out by the USEPA revealed atrazine as the common herbicide contaminating the well water (Ritter, 1990; Quackenboss et al., 2000; Focazio et al., 2006). Meanwhile, up to 40% of surface water in 31 states and 10% of groundwater in 13 states had earlier tested positive for atrazine (US EPA, 1989). On the other hand, out of 351 hand-dug wells monitored for atrazine on Ontario farms by the Ontario Ministry of Environment in Canada, 17% of the hand-dug wells recorded atrazine concentrations that were above 1 μg/L (Ontario Ministry of the Environment, 1987). This later prompted Canada to establish a safe limit of 5 μg/L for atrazine in drinking water (Health Canada, 1993). Up till now, no study has been conducted in some rural agricultural areas in Nigeria to monitor atrazine in drinking water from hand-dug well and surface waters as well as its potential health implications.

Although the toxicological profiles of atrazine in experimental animals and human studies have been widely documented (US EPA, 2007; WHO, 2011). Of major interest is its potential to induce reproductive dysfunction in animals. Atrazine has been reported to negatively alter some reproductive hormones in animals (Stoker et al., 2000; Zirkin et al., 2001; Hayes, 2004; Swan, 2006; WHO, 2011) and decrease fertility in rats at 120 mg/kg/day of exposure (Simic et al., 1994; McElroy et al., 2000). Disruptions of plasma and gonadal sex steroids in fish exposed to atrazine were observed (Spano et al., 2004). Furthermore, atrazine induced gonadal-histo-functional alteration in crocodilian reptiles (Rey et al., 2009) and spermatogenesis disruption in birds (Hussain et al., 2011). Abarikwu et al. (2010) studied changes in sperm characteristics and oxidative stress parameters in the testis and epididymis of rats exposed to atrazine at 0, 100, and 200 mg/kg body weight for 7 and 16 days. The herbicide was observed to impair reproductive function and elicits a depletion of the antioxidant defence system in the testis and epididymis, indicating the induction of oxidative stress (Abarikwu et al., 2010). Although studies have documented the reproductive toxicity of atrazine in animals, there is a need to ascertain this toxicity by considering environmentally relevant concentrations found in drinking water from rural agricultural communities to which the inhabitants may be directly exposed.

Moreover, ensuring sustainable access to clean and safe water is one of the major targets of the 2030 Agenda for Sustainable Development Goals (SDGs) (WHO, UNICEF, 2017), and achieving this target will involve significant improvements in rural areas of Sub-Saharan Africa, which have been denied access to safe and clean drinking water (Sogbanmu et al., 2020). More than one-third of the Nigerian population has been denied access to quality drinking water sources due to uncontrolled anthropogenic activities (WHO, UNICEF, 2017; Sogbanmu et al., 2020). Therefore, the majority of Nigerians in rural communities (where there are higher agricultural activities) still depend on streams, rivers, and hand-dug wells for their drinking water. In the year 2007, the governing council of the Standard Organization of Nigeria established a maximum permissible level of 0.02 mg/L for 2, 4, 6—trichlorophenol in drinking water (Standards Organization of Nigeria, 2007) but provided no limits for the widely used atrazine herbicide.

We designed this study to 1) determine the residue of atrazine in water from the rural communities of Ijebu-North LG, Southwest Nigeria. 2) Evaluate the potential human health risk associated with exposure to atrazine 3) carry out reproductive toxicity of atrazine concentrations found in the water from the study area.

## Materials and methods

### Study area

This study was carried out in Ijebu-North LGA of Ogun State Nigeria because agriculture is the economic mainstay of the LGA. The local government comprises six communities including Ago-Iwoye, Ijebu-Igbo, Oru, Ilaporu, Awa, and Mamu with a major farm village in each community. The LGA has a 2016 population projection of 390, 200 (National Population Commission, 2016) and a total land area of 967 km^2^. Ijebu North LGA is bounded by Oluyole LGA of Oyo State in the North, Ijebu East L.G in the west, Odogbolu and Ijebu Ode L.G. in the south, and Ikenne L.G. in the east.

### Water sample collection

**FIGURE 1:**
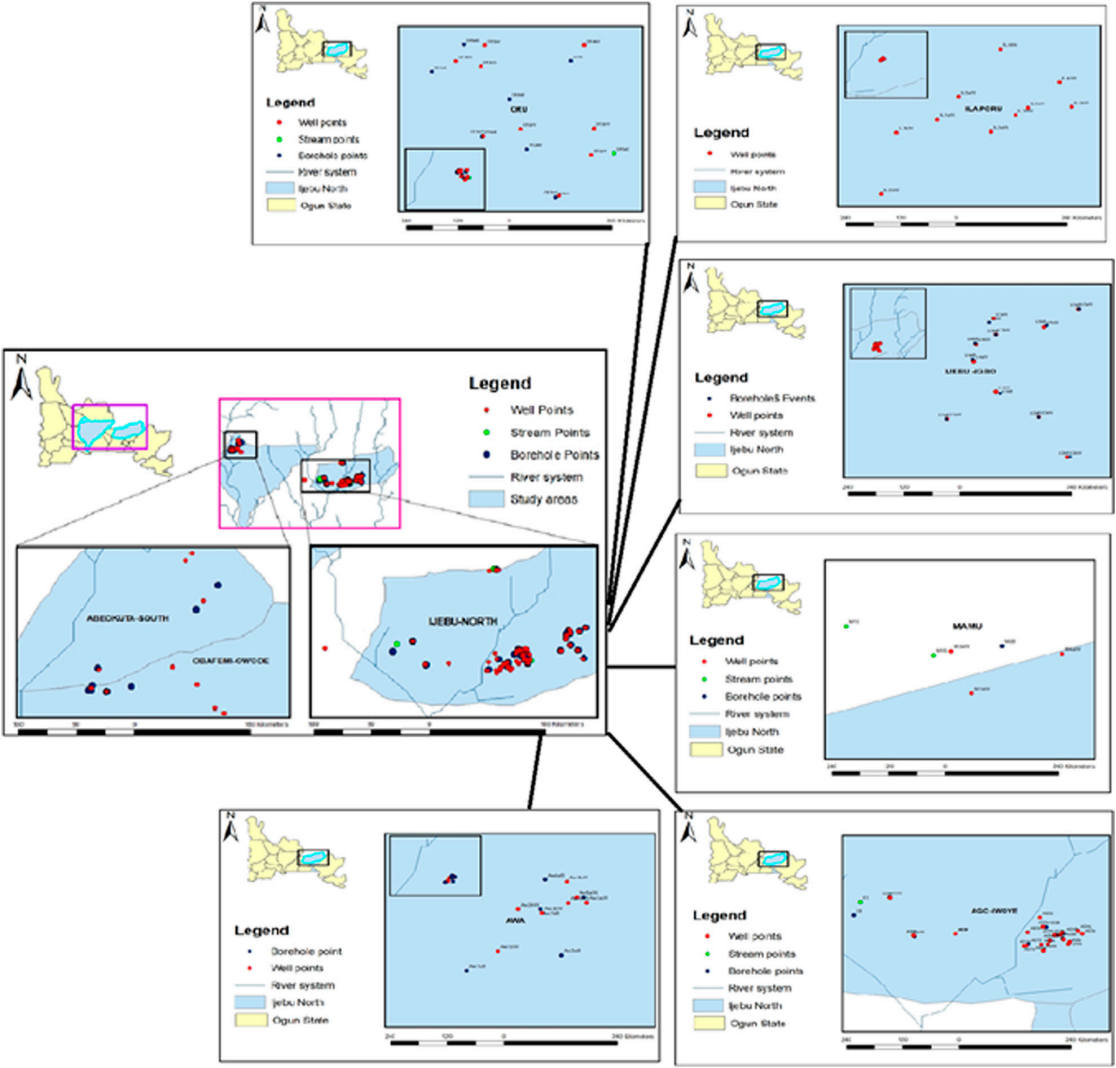
Map of each community and Ijebu-North showing the sampling point.

Hand-dug well (HDW), borehole (BH), and stream waters from each of the communities in Ijebu-North (Figure 1) were collected in 20 ml amber glass bottles according to the procedures of the United States Geological Survey (Koterba et al., 1995). We also collected waters from HDW and BH from the state capital, Abeokuta urban area and this serves as the control location. The number of HDW, BH, and streams sampled from the community is shown in Supplementary Table S1. In total, 69 HDW, 40 BH, and four streams were sampled. The water sample was collected from the farm settlement and collection was maintained within at least a 400-m distance from each sampling point throughout the community. The depths of all the HDW and the coordinates of each sampling point are shown in Supplementary Tables S2A-G. The depth of all the HDWs sampled was less than 12 m. However, the depths of the boreholes could not be measured. All the water samples were filtered at the point of collection and transported on ice to the laboratory for atrazine concentration analysis. Values obtained were used for the evaluation of human health risks posed by the herbicide. The highest concentration of atrazine observed in a water sample from each of the communities was administered to experimental rats in a sub-chronic hepatotoxicity study.

### Determination of atrazine residue

The residue of atrazine was determined in the water sample by using the approved protocols for drinking water monitoring of contaminants (Huang et al., 2003; US EPA, 2009) with few modifications. A stock standard of atrazine was prepared by weighing 10 mg of analytical standard (Sigma-Aldrich, St. Louis, MO, United States ) into a 100 ml flask, diluted to the mark with methanol, and stored at −18°C in the dark. Solid-Phase Extraction (SPE) technique was employed with a C18 SPE cartridge, which was conditioned under gravity with 10 ml each of methanol and deionized water. Another 2 ml of deionized water was added to avoid dryness and the valve was closed to the vacuum manifold. The configuration was completed by attaching a 75-ml solvent reservoir to the top of the C18 cartridge that was connected to the manifold. The water sample (200 ml, pH adjusted to 6) was transferred to the reservoir and pumped through the cartridge at a flow rate of 10 ml/min. The cartridge was later washed with 20 ml of deionized water. The cartridge was subjected to a vacuum system for 25 min to remove water and then, totally dried with a stream of nitrogen gas. The elution of the compound retained was done with 2 ml of methanol and the organic extract was completely dried with a rotary evaporator at 40°C and a stream of nitrogen gas. The final sample was reconstituted in acetone to a final volume of 1.0 ml that is ready for GC-MS analysis. The analysis was performed on GC (Agilent Technologies, Model; 7890A) with Mass Selective Detector (Model; 5975C) and the analytical procedures follow the USEPA good laboratory practice standards (40 CFR Part 79.60, 1994). The initial oven temperature was programmed at 110°C for 2 min at 10°C/minute to 180°C at 20°C/minute to 250°C for 8 min. The electron ionization (70v) and ion source temperature (230°C) were also programmed. A total of 1µ/L of the sample was injected and Agilent technologies HP5MS column (30 m × 0.25 mm X 0.320 µm) was used for separation. Helium gas was used as the carrier gas at 65 psi. Quantification and identification of the peak areas were done by comparison with the standard used to prepare the calibration standard solution.

### Human health risk evaluations

We carried out non-carcinogenic human health risks for adults and children through ingestion and dermal routes in this study. The health risk was evaluated following the standard protocol (EPA, 1989; Resources, 2006; Health Canada, 2010; Almasi et al., 2020; Dehghani et al., 2022). The average daily exposure concentration known as average daily intake (ADI) was computed using Eqs 1, 2 as below

$${\mathrm{ADI}}_{\mathrm{ingestion}}=\frac{CW\;x\;IRW\;x\;EF\;x\;ED}{BW\;x\;AT}$$ (1)

where CW- atrazine level in water (mg/L); IRW- ingestion rate of water (L); EF- exposure frequency (days/year); ED-exposure duration (years); BW -body weight (kg); AT-average exposure time (days) (EPA, 1989; Resources, 2006; Health Canada, 2010; Almasi et al., 2020; Dehghani et al., 2022).

$${\mathrm{ADI}}_{\mathrm{dermal}}=\frac{CW\;x\;KP\;x\;EF\;x\;SA\;x\;ET\;x\;ED\;x\;CP}{BW\;x\;AT}$$ (2)

SA-skin area (cm^2^), KP- coefficient for dermal permeability (cm/h), ET-exposure time (hrs/day), CF- conversion factor (mg/kg) (EPA, 1989; Resources, 2006; Health Canada, 2010; Almasi et al., 2020; Dehghani et al., 2022).

The hazard quotient (HQ) of atrazine via ingestion and dermal routes was estimated with the standard United States EPA reference dose (RfD) (0.035 mg/kg/day) shown in Equation 3 below

$$HQ=\frac{ADI}{RfD}$$ (3)

The hazard index (HI) of atrazine exposure for adults and children was estimated through the summation of the HQ obtained for ingestion and dermal contact.

## Toxicity study

### Experimental animal and study design

A total of thirty (Huang et al., 2003) male albino rats (140 ± 10 g) were randomized into five groups (four treatments and one control) of six rats/group and orally exposed to atrazine (PESTANAL, Sigma Aldrich St Louis, United States ; purity 98.2%) at 0.01 mg/L, 0.03 mg/L, 0.04 mg/L, and 0.08 mg/L concentrations. Atrazine was diluted in a watery suspension and orally administered, with gavage, to the rats once a day in a volume of 0.25 ml/100 g of body weight as previously described (Romano et al., 2010; Owagboriaye et al., 2017). Distilled water was administered to the control group and the exposure scenario lasted for 12 weeks. The concentrations were selected because they were the highest values of atrazine recorded in the water from each of the communities. We handled the rats by following the guidelines of the local ethical committee in the Animal Care Unit (ACU) of Olabisi Onabanjo University as well as ethical guidelines (regulation CEE 86/609).

### Collection of samples and preparation

Samples were collected and prepared according to the methods described in Owagboriaye et al. (2022). Retro orbital sinus technique with the microhematocrit tube was used to collect blood samples into a plain sample tube and serum was separated within 1 h after collection. The testis was excised and rinsed in saline solution. The excised testis was divided into two portions. A portion was homogenized in 10%, w/v phosphate buffer (pH 7.4), centrifuged and the supernatant was used for the biochemical assays. The second portion was fixed in 10% neutral-buffered formalin for histopathological investigations. The rat’s epididymis was dissected and sperm cells were collected for sperm parameters analysis.

### Hormonal assay

Enzyme-Linked immunosorbent Assay (ELISA) kit (Bio check, United States ) was used to determine the concentration of Follicle Stimulating Hormone (FSH), testosterone, prolactin, and Luteinizing hormone (LH) and the protocols follow the manufacturer’s guide.

### Estimation of ROS and oxidative stress parameters in the testis

The level of ROS in the testicular tissue was determined by incubating 100 μL of an aliquot from testicular homogenate with 5 μL 2′,7′-dichloro-dihydro fluorescein diacetate (DCFH-DA) at 37°C for 60 min as previously described in Pérez-Severiano et al. (2004) and Owagboriaye et al. (2021), Owagboriaye et al. (2022). The thiobarbituric acid reactive substance (TBARS) assay described in Okhawa et al. (1979), which involves the measurement of malondialdehyde (MDA) was employed for the determination of lipid peroxidation. The activity of superoxide dismutase (SOD) was determined according to Asada et al. (1974). The amount of enzyme needed to yield 50% inhibition of nitroblue tetrazolium (NBT) per minute indicates a unit of SOD. The method of Hassan and Barakat (2008) was followed for the determination of reduced glutathione (GSH) concentration. The activity of catalase was determined by monitoring the disappearance of hydrogen peroxide at 240 nm (Aebi, 1984). A decrease of 1 µmol in a hydrogen peroxide/minute denotes one unit of the enzyme. We adopted the protocol of Necheles et al. (1968) for the determination of GPx activity using hydrogen peroxide and GSH as substrates. A total of 1 µg of GSH consumed/minute indicates a unit of GPx. The standard protocol of Habig et al. (1974) was adopted for the determination of GST activity. The method of Lowry et al. (1951) was used for protein determination and the expressions of all the antioxidant enzyme activities are in units per milligram of protein.

### Estimation of the testis membrane-bound ATPase enzyme

The amount of phosphorus liberated from the incubation mixture of the testis homogenate and 5 mM of ATP, 60 mM of NaCl, 2 mM of MgCl_2_, 20 mM of KCl, 2 Mm of CaCl_2_, and protein enzyme was estimated for the determination of Ca^2+^ATPase, Na^+^/K^+^ATPase and Mg^2+^ATPase activities (Dedeke et al., 2018; Owagboriaye et al., 2022). We also set up control through the addition of enzyme after trichloroacetic acid at the end of the incubation period. We adopted the established protocol of Lowry et al. (1951) for protein estimation.

### Epididymis sperm parameter assessment

Assessments of sperm count and percentage motility were carried out according to the protocol described in Hassan and Barakat (2008). Epididymides contents were dissected into 10 ml of 0.87% normal saline and dropped on clean slides. The movement or percentage motility of the sperm cells was determined using Eq. 4 as below

$$\%\;motility=\frac{number\;of\;sperm\;cells\;with\;progressive\;movements}{200}\times 100\%$$ (4)

The sperm cells were also counted using a Hausser Scientific haemocytometer (Hausser Scientific 3,520, Horsham, PA, United States ). Sperm abnormality assessment was done according to the modified method of Bakare et al. (2005). Epididymal sperm cells were suspended in normal saline and 1% eosin Y stain. A smear was prepared on slides, which were allowed to air-dry and coded for subsequent microscopic examination. A total of 1,000 sperm cells were assessed for morphological abnormalities for each rat.

### Histopathological examinations

The testis excised was routinely processed for histopathological investigation, sectioned at 4-5 thick, stained with Hematoxylin-Eosin stain, and mounted in a neutral DPX medium as previously described (Owagboriaye et al., 2022). The prepared slides were viewed at ×400 magnifications.

### Statistical methods

IBM Statistical Package (SPSS) version 20.0 was used to analyze the data obtained. We compared the mean values with the Analysis of Variance (ANOVA) and the results were presented as Mean ± Standard Error of Mean (SEM). Student-Newman-Keuls (SNK) was used for the post hoc test and a Probability value less than 0.05 was considered to be statistically significant.

## Results

### Level of atrazine in water from Ijebu-North

Residues of atrazine in hand-dug wells, boreholes, and streams water from the six communities in Ijebu-North and Abeokuta are shown in Table 1. A total of 41 hand-dug wells, 22 boreholes, and all four streams showed varying concentrations of atrazine. Ago-Iwoye recorded the highest concentration of 0.08 mg/L in its hand-dug well water. This is followed by well water from Awa and Mamu (0.04 mg/L). Ijebu-Igbo and Oru recorded the highest atrazine concentration of 0.03 and 0.01 mg/L respectively. Atrazine was not detected in borehole water from Abeokuta.

**TABLE 1:** Concentration of atrazine (mg/L) in water from the communities in Ijebu-North Local Government of Ogun State, Nigeria.

	Abeokuta		Ijebu-Igbo		Oru			Ilaporu	Awa		Ago-iwoye			Mamu		
Point	Well	BH	Well	BH	Well	BH	Stream	Well	Well	BH	Well	BH	Stream	Well	BH	Stream
SP1	ND	ND	0.03	0.01	0.01	0.005	0.004	0.01	0.04	0.01	0.08	0.01	0.003	0.04	0.003	BDL
SP2	ND	ND	0.03	0.003	0.01	BDL		ND	0.02	BDL	0.03	BDL		0.03		0.003
SP3	BDL	ND	ND	ND	0.007	ND		0.003	0.04	0.01	0.02	0.003		0.01		
SP4	ND	ND	0.01	ND	ND	0.004		BDL	0.03	0.01	0.03	BDL				
SP5	ND	ND	0.02	0.003	ND	ND		ND	0.04	BDL	0.02	BDL				
SP6	BDL	ND	ND	ND	ND	ND		0.01	ND		0.02	0.003				
SP7	ND	ND	ND	BDL	0.01	0.003		0.003	0.03		0.01	BDL				
SP8	BDL	ND	ND	ND	0.004			ND			0.02					
SP9	ND	ND	0.02	0.003	ND			ND			ND					
SP10	ND	ND	ND	ND				ND			0.02					
SP11											ND					
SP12											0.01					
SP13											0.01					
SP14											ND					
SP15											0.01					
SP16											0.004					
SP17											ND					
SP18											0.003					
SP19											ND					
SP20											ND					

SP-sampling point; ND-not, detected; BH- borehole; BDL-below detection limit.

### Human health risk evaluation

**FIGURE 2:**
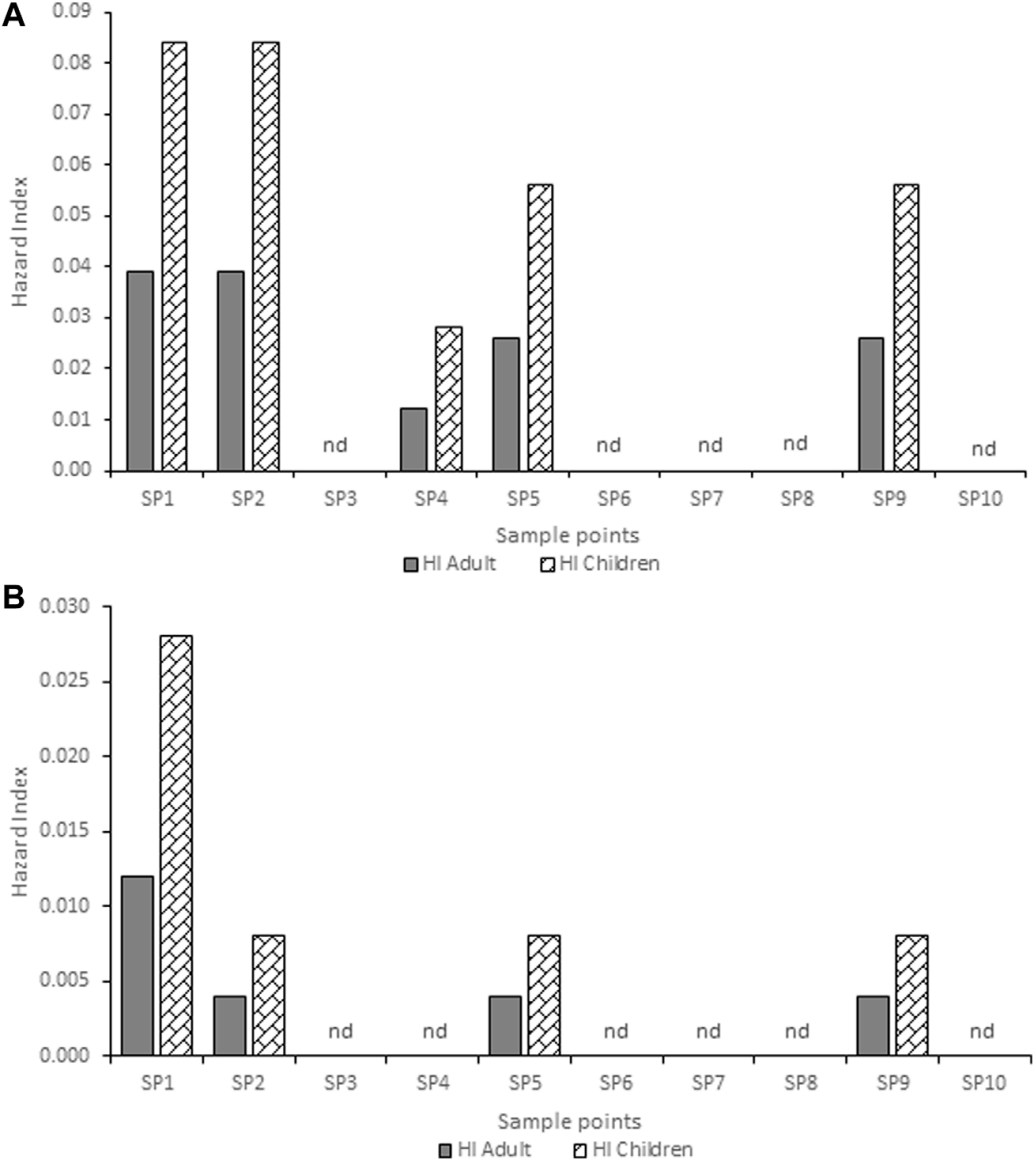
Hazard index for adults and children associated with exposure to atrazine concentration in **(A)** hand-dug well **(B)** borehole water from Ijebu-Igbo community. SP-Sampling point; nd-non-detected.

**FIGURE 3:**
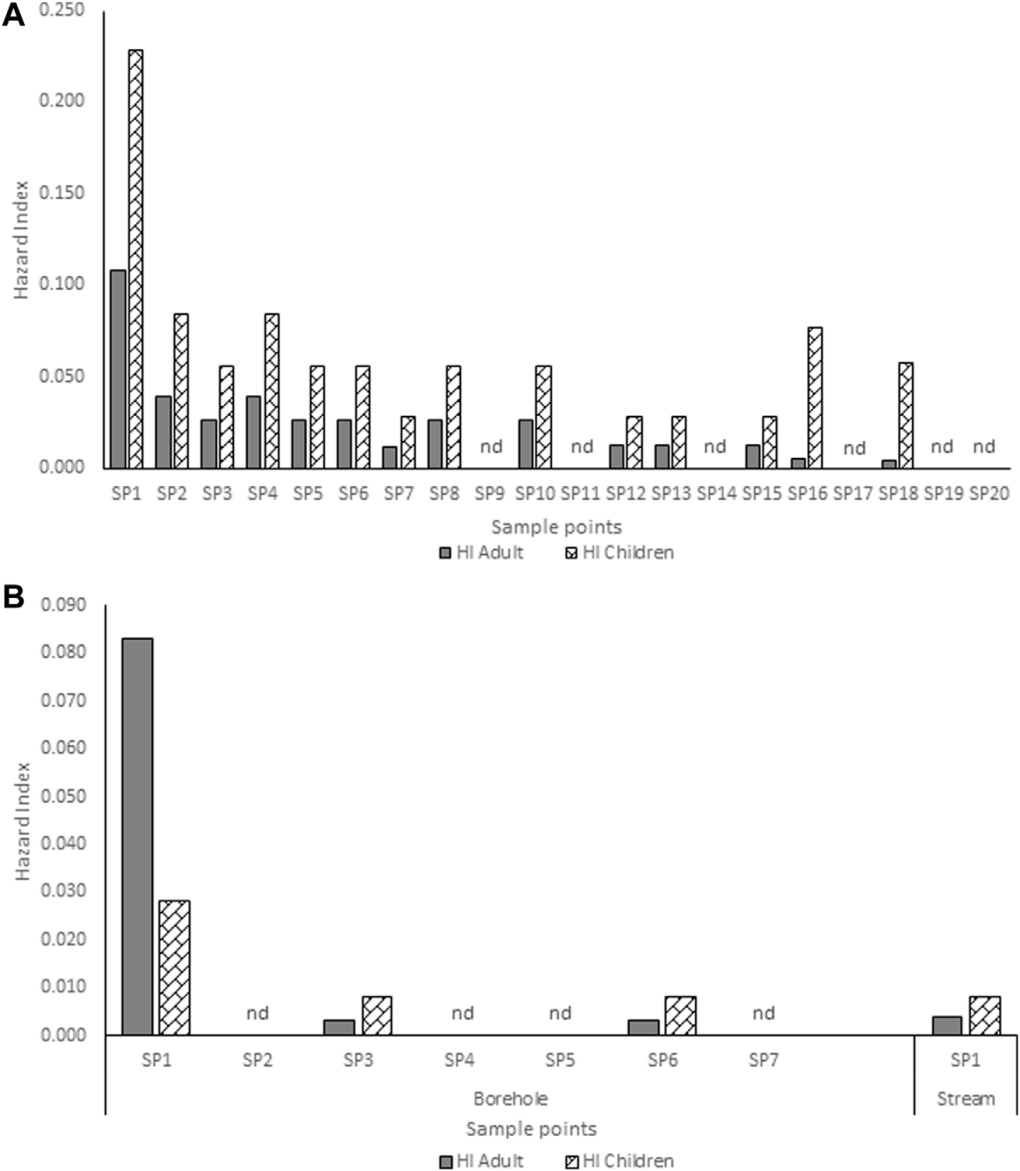
Hazard index for adults and children associated with exposure to atrazine concentrations in **(A)** hand-dug well **(B)** borehole and stream water from Ago-Iwoye community. SP-Sampling point; nd-non-detected.

The estimated HQ for children and adults according to the atrazine concentrations detected in each community are shown in Supplementary Tables S3,4,5,6,7. Ago-Iwoye recorded the highest HQ values of 0.146 and 0.063 for ingestion in children and adults, and HQ values of 0.082 and 0.045 for dermal contact in children and adults respectively. This was followed by the Awa community where the HQ values of 0.074 and 0.016 for ingestion in children and adults, and HQ values of 0.040 and 0.023 for dermal contact in children and adults respectively were observed. The estimated HI for children and adults according to the atrazine concentrations detected in Ijebu-Igbo and Ago-Iwoye, Oru, Awa, Mamu and Ilaporu are shown in Figures 2, 3, respectively. However, the estimated HI for children and adults according to the atrazine concentrations detected in Oru, Awa, Mamu and Ilaporu are shown in Supplementary Figures S1,S2,S3,S4 The HI value in children ranges from 0.020 to 0.228 while the value ranges from 0.004 to 0.108 for adults. However, the values were less than one, which is the standard acceptable value.

### Levels of serum testosterone and prolactin hormones in the experimental rats

**FIGURE 4:**
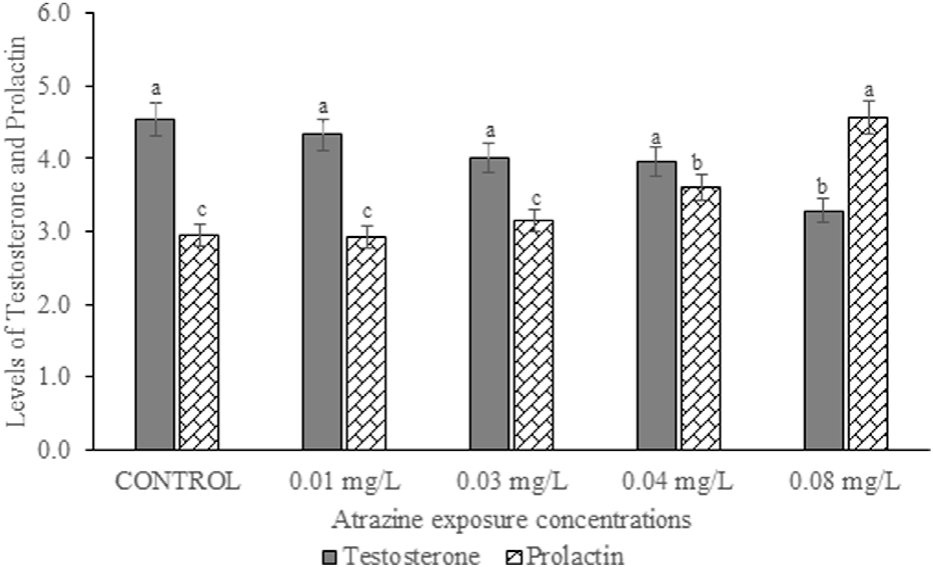
Levels of serum testosterone (ng/ml) and prolactin hormones (ng/ml) of male albino rat exposed to atrazine concentrations in drinking water from Ijebu-North, Southwest Nigeria; Error bars represent standard deviation; abc = bars with similar alphabets are not significantly different between the concentration (*p* > 0.05).

Levels of serum testosterone and prolactin hormones of male albino rat exposed to atrazine concentrations in drinking water from Ijebu-North, Southwest Nigeria is presented in Figure 4. Results showed a reduction in the serum level of testosterone hormone with an increase in the concentration of atrazine exposure. However, the reduction was only significant (*p* < 0.05) in rats exposed to 0.08 mg/L concentration of atrazine compared to control and those exposed to 0.01, 0.03, and 0.04 mg/L concentrations of atrazine. On the other hand, the level of serum prolactin was observed to significantly (*p* < 0.05) increase in the experimental animals exposed to atrazine at 0.04 and 0.08 mg/L concentrations. However, there was no significant (*p* > 0.05) difference in the level of serum prolactin hormone in the control rats and those exposed to 0.01 mg/L and 0.03 mg/L concentrations of atrazine.

### Levels of follicle-stimulating and luteinizing hormones in the experimental rats

**FIGURE 5:**
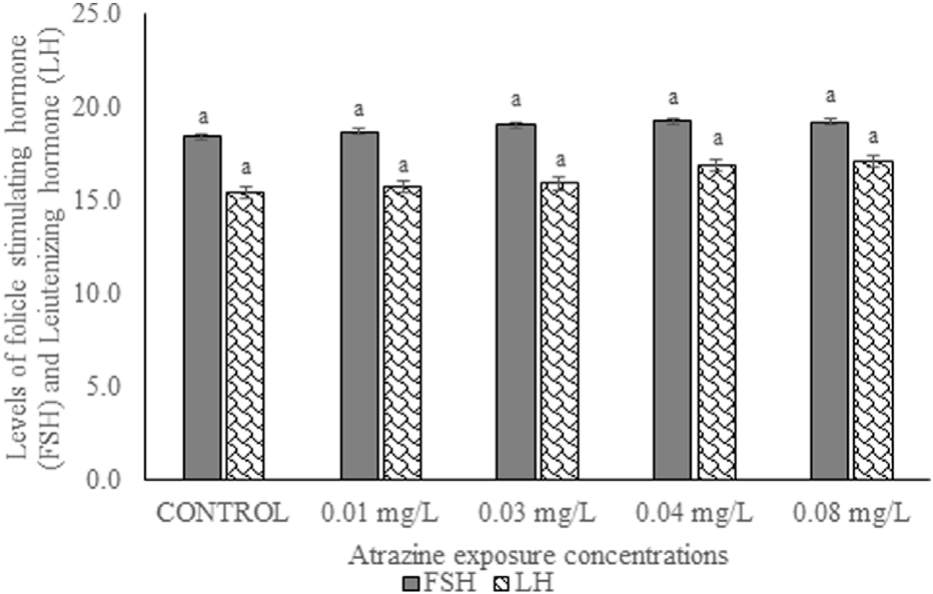
Levels of follicle-stimulating (mlu/mL) and luteinizing hormones (mlu/mL) of male albino rat exposed to atrazine concentrations in drinking water from Ijebu-North, Southwest Nigeria; Error bars represent standard deviation; abc = bars with similar alphabets are not significantly different between the concentration (*p* > 0.05).

The serum levels of FSH and LH of male albino rat exposed to atrazine concentrations in drinking water from Ijebu-North, Southwest Nigeria is shown in Figure 5. Although the level of the gonadotropic hormone was higher in all the rats exposed to the varying concentrations of atrazine, not significant (*p* > 0.05) compared to control.

## Antioxidant enzymes activities in the testicular tissue of the experimental rats

Antioxidant enzyme activities and concentration of GSH in the testicular tissue of male albino rats exposed to atrazine concentrations in drinking water from Ijebu-North, Southwest Nigeria are shown in Table 2. A significant (*p* < 0.05) increase in SOD activity was observed in the testicular tissue of rats exposed to 0.03 mg/L and 0.04 mg/L concentrations of atrazine compared to other groups. On the other hand, the concentration of GSH was observed to significantly (*p* < 0.05) reduce in the experimental rats with an increase in the concentration of atrazine exposure. In a similar trend, CAT activity was significantly (*p* < 0.05) reduced only in rats exposed to a 0.08 mg/L concentration of atrazine. However, there was no significant difference (*p* > 0.05) in the CAT activity in the control rats and those exposed to 0.01, 0.03, and 0.04 mg/L concentrations of atrazine. Also, the activity of GPx observed in the control rats was not significantly different (*p* > 0.05) from that observed in the rats exposed to 0.01 mg/L and 0.03 mg/L concentrations of atrazine. However, GPx activity was significantly reduced (*p* < 0.05) in the rats exposed to a 0.08 mg/L concentration of atrazine.

**TABLE 2:** Antioxidant enzymes activities in the testicular tissue of male albino rat exposed to atrazine concentrations in drinking water from Ijebu-North, Southwestern Nigeria.

Treatments	SOD (U/mg protein)	GSH (U/g tissue)	CAT (U/mg protein)	GPx (U/mg protein)
Control	6.96 ± 1.49^c^	11.56 ± 2.22^a^	13.33 ± 0.75^a^	19.74 ± 1.12^a^
0.01 mg/L	8.98 ± 1.26^b^	10.17 ± 0.69^b^	12.19 ± 1.49^a^	19.37 ± 0.82^a^
0.03 mg/L	10.24 ± 0.81^a^	8.85 ± 0.47^c^	13.04 ± 7.32^a^	18.15 ± 0.72^a^
0.04 mg/L	10.50 ± 0.99^a^	8.69 ± 0.51^c^	13.16 ± 1.57^a^	16.27 ± 1.27^b^
0.08 mg/L	8.35 ± 0.42^b^	7.76 ± 0.48^c^	11.29 ± 1.26^b^	10.32 ± 1.88^c^

a Mean values (±Standard deviation) in the same column having similar superscripts are not significantly different (*p* > 0.05); SOD, Superoxide dismutase; GPx, Glutathione peroxidase; GSH, Reduced glutathione

b Mean values (±Standard deviation) in the same column having similar superscripts are not significantly different (*p* > 0.05); SOD, Superoxide dismutase; GPx, Glutathione peroxidase; GSH, Reduced glutathione

c Mean values (±Standard deviation) in the same column having similar superscripts are not significantly different (*p* > 0.05); SOD, Superoxide dismutase; GPx, Glutathione peroxidase; GSH, Reduced glutathione

### Levels of reactive oxygen species (ROS) and lipid peroxidation in the testicular tissue

**FIGURE 6:**
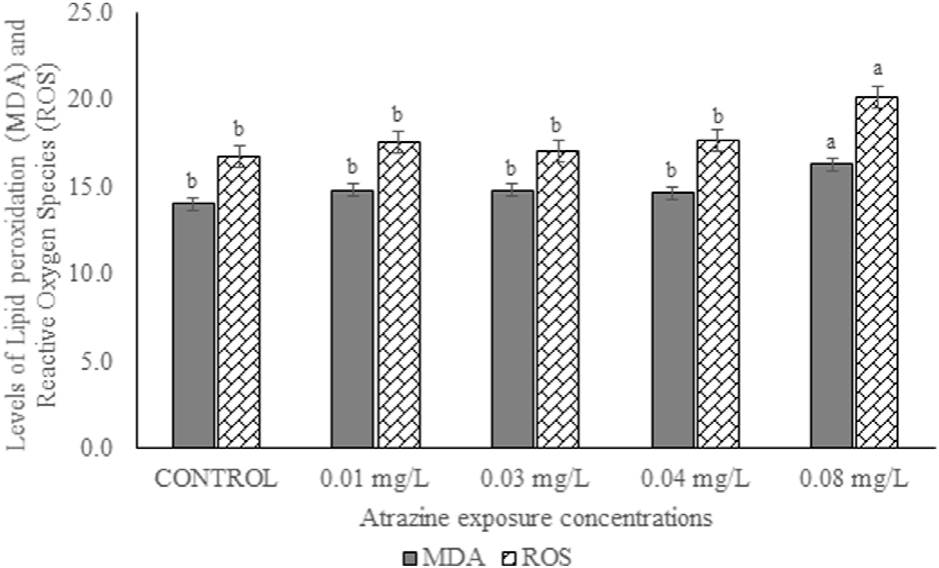
Levels of ROS (nmoles/DCF/g tissue/min)) and MDA (nmol/g tissue) in the testes of male albino rat exposed to atrazine concentrations in drinking water from Ijebu-North, Southwest Nigeria; Error bars represent standard deviation; abc = bars with similar alphabets are not significantly different between the concentration (*p* > 0.05).

Levels of ROS and MDA in the testicular tissue of male albino rats exposed to atrazine concentrations in drinking water from Ijebu-North, Southwest Nigeria are shown in Figure 6. Although ROS generation and MDA levels were observed to increase with an increase in atrazine concentration, the levels of ROS and MDA recorded in the control rats were not significantly (*p* > 0.05) different from those recorded in the rats exposed to 0.01 mg/L, 0.03 mg/L and 0.04 mg/L concentrations of atrazine. However, levels of ROS and MDA were significantly (*p* < 0.05) increased in the rats exposed to 0.08 mg/L concentrations of atrazine.

### Activities of glutathione-S-transferase in the testicular tissue

**FIGURE 7:**
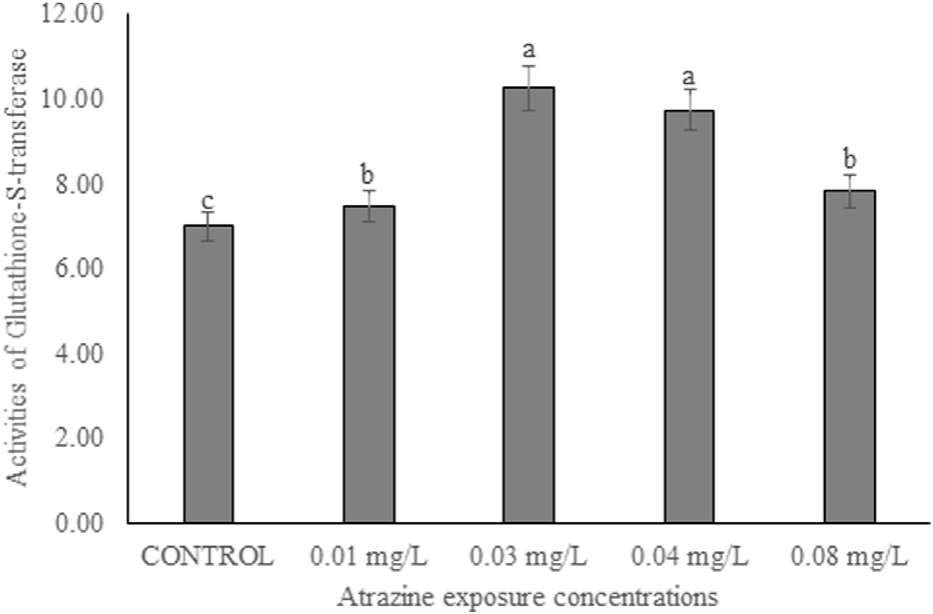
Activities of glutathione-S-transferase (U/mg protein) in the testicular tissue of male albino rat exposed to atrazine concentrations in drinking water from Ijebu-North, Southwestern Nigeria.

The activity of GST in the testicular tissue of male albino rats exposed to atrazine concentrations in drinking water from Ijebu-North, Southwest Nigeria is shown in Figure 7. The enzyme activity was observed to significantly (*p* < 0.05) increase in all the rats exposed to varying concentrations of atrazine compared to control. GST activity was significantly increased in the testicular tissue of rats exposed to 0.03 and 0.04 mg/L concentrations of atrazine. But there was no significant difference (*p* > 0.05) in the enzyme activity in the testis of rats exposed to 0.01 and 0.08 mg/L concentrations of atrazine.

### Activities of membrane-bound ATPase enzymes in the testis of albino rats

**FIGURE 8:**
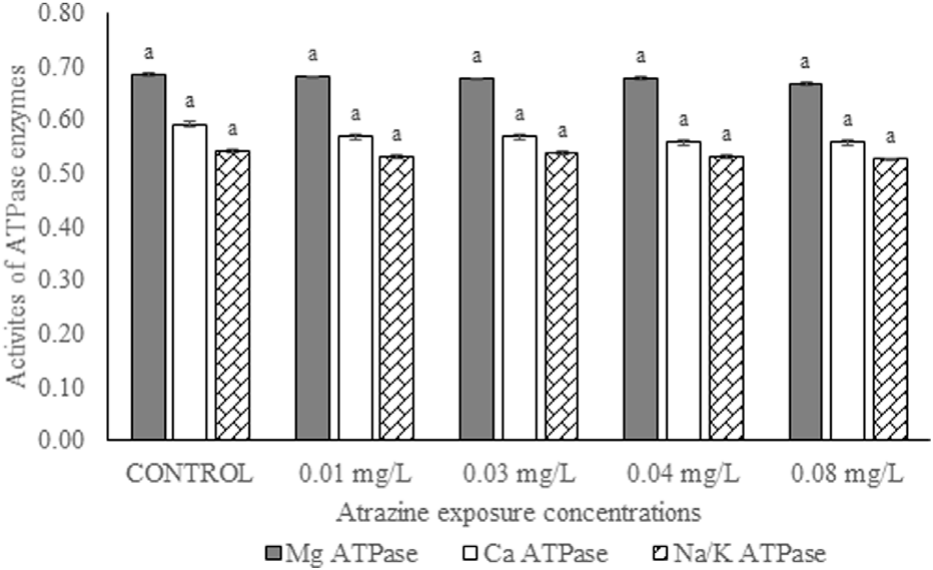
Activities of ATPase enzymes (µg pi liberated/min/mg protein) in the testis of male albino rats exposed to atrazine concentrations in drinking water from Ijebu-North, Southwest Nigeria; Error bars represent standard deviation; abc = bars with similar alphabets are not significantly different between the concentration (*p* > 0.05).

Activities of membrane-bound ATPase enzymes in the testis of albino rats exposed to atrazine concentrations in drinking water from Ijebu-North, Southwest Nigeria are represented in Figure 8. Although the activity of Ca^2+^ ATPase was highest in the control rats, there were no significant (*p* > 0.05) differences in the activities of Ca^2+^ ATPase, Mg^2+^ ATPase, and Na^+^/K^+^ ATPase in the control rats and those exposed to 0.01, 0.03, 0.04 and 0.08 mg/L concentrations of atrazine.

### Sperm motility and sperm count of the experimental rat

**FIGURE 9:**
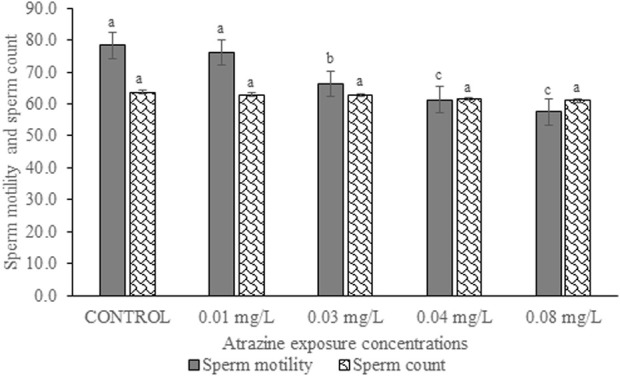
Sperm motility (%) and sperm count (×10^6^ cells/ml) of rats exposed to atrazine concentrations in drinking water from Ijebu-North, Southwest Nigeria; Error bars represent standard deviation; abc = bars with similar alphabets are not significantly different between the concentration (*p* > 0.05).

Sperm motility and sperm count of experimental rats exposed to atrazine concentrations in drinking water from Ijebu-North, Southwest Nigeria are shown in Figure 9. Percentage sperm motility was observed to significantly (*p* < 0.05) reduce in the experimental rats with an increase in the concentration of atrazine exposure. Sperm motility observed in control rats was not significantly (*p* > 0.05) different from that of rats exposed to a 0.01 mg/L concentration of atrazine. Also, the sperm motility observed in rats exposed to 0.04 mg/L and 0.08 mg/L atrazine concentration was not significantly (*p* > 0.05) different. Although sperm count was observed to reduce in the experimental rats with an increase in the concentration of atrazine exposure, the reduction was not significant (*p* > 0.05) compared to control.

### Sperm morphology of male albino rat

**FIGURE 10:**
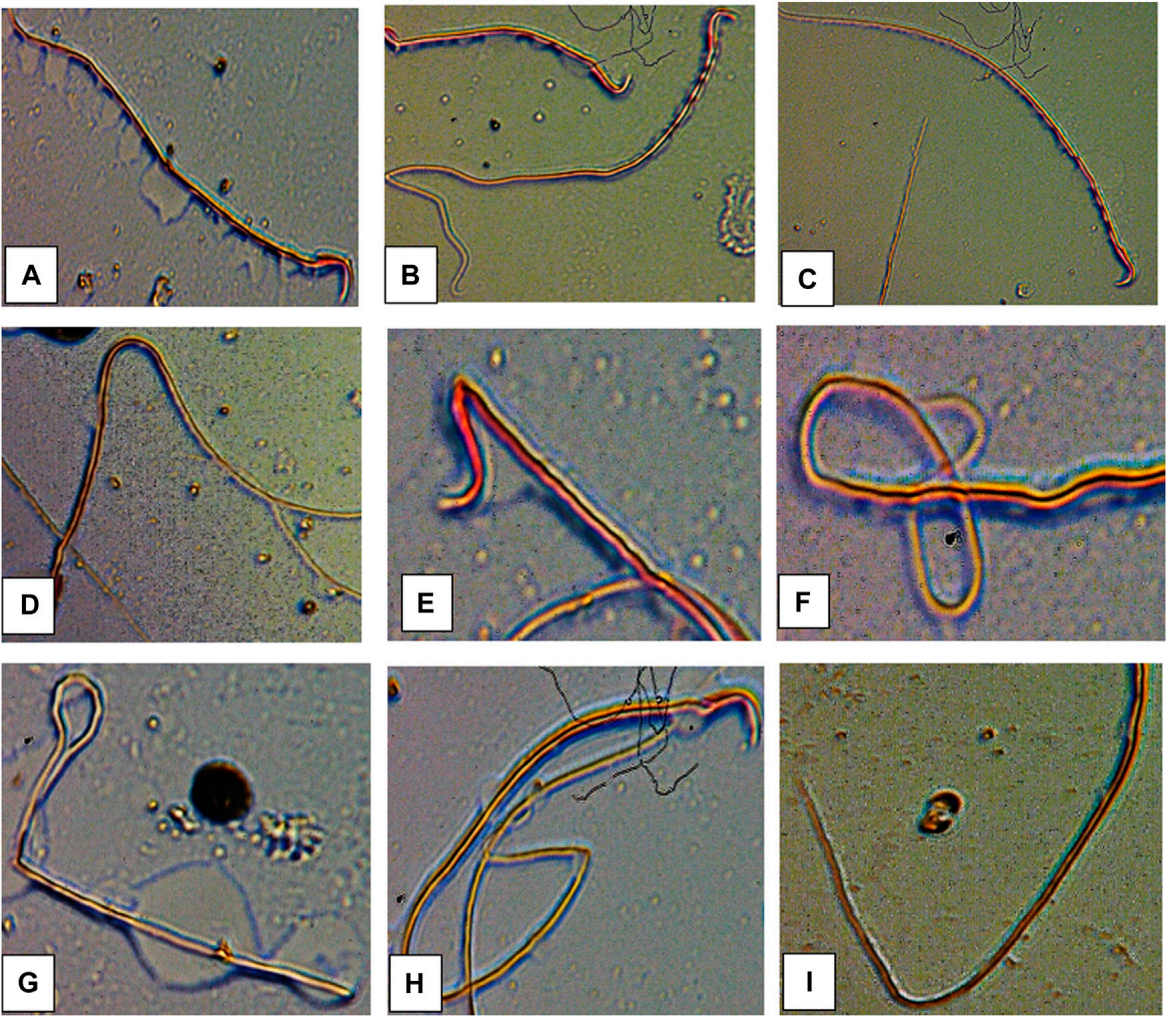
Abnormal sperm cells induced in rats exposed to atrazine **(A)** normal sperm cell **(B)** sperm with abnormal tail **(C)** normal sperm cell **(D)** sperm with bent body **(E)** sperm with damaged head **(F)** sperm with bent tail **(G)** bent tail with damaged head **(H)** banana shape **(I)** bent tail.

The results of sperm abnormality assessment in rats exposed to atrazine concentrations in drinking water from Ijebu-North, Southwest Nigeria are displayed in Table 3 and Figure 10. Total sperm abnormality and percentage abnormality were significantly (*p* < 0.05) increased in rats exposed to atrazine at 0.08 mg/L concentration. Also, sperm abnormalities were observed to significantly (*p* < 0.05) increase in the experimental rats with an increase in the concentration of atrazine exposure. However, the number of sperms with abnormal tails was observed to significantly contribute to the total abnormal sperm of the experimental rats. On the other hand, there were no significant (*p* > 0.05) differences in the number of sperms without hook, sperms with damaged head, and banana-shaped sperms recorded in the control rats and those exposed to 0.01, 0.03, 0.04, and 0.08 mg/L concentrations of atrazine.

**TABLE 3:** Sperm morphology of male albino rat exposed to atrazine concentrations in drinking water from Ijebu-North, Southwestern Nigeria.

Parameters	Control	0.01 mg/L	0.03 mg/L	0.04 mg/L	0.08 mg/L
Abnormal Tail	10.40 ± 1.52^d^	10.80 ± 1.48^d^	31.20 ± 2.59^c^	40.20 ± 1.92^b^	45.20 ± 4.49^a^
Without head	0.20 ± 0.45^c^	0.80 ± 0.84^b^	1.20 ± 1.30^b^	1.60 ± 1.14^b^	2.60 ± 1.14^a^
Damaged Head	0.80 ± 0.84^a^	1.00 ± 0.71^a^	1.40 ± 0.89^a^	1.40 ± 0.89^a^	1.60 ± 0.55^a^
Without hook	0.80 ± 0.84^a^	1.00 ± 0.71^a^	1.20 ± 0.84^a^	1.60 ± 0.55^a^	1.60 ± 0.55^a^
Banana shaped	1.40 ± 1.^a^	2.20 ± 0.45^a^	2.80 ± 1.48^a^	3.20 ± 1.30^a^	3.60 ± 1.52^a^
Without Tail	1.00 ± 1.22^d^	1.00 ± 0.71^d^	9.80 ± 3.70^c^	21.00 ± 3.16^b^	25.00 ± 2.24^a^
Total Abnormal	14.60 ± 2.30^d^	16.80 ± 2.59^d^	47.60 ± 1.95^c^	70.00 ± 4.85^b^	77.60 ± 6.07^a^
% Abnormality	1.46 ± 0.23^d^	1.68 ± 0.26^d^	4.76 ± 0.19^c^	7.00 ± 0.48^b^	7.76 ± 0.61^a^

a Mean values (±Standard deviation) in the same column having similar superscripts are not significantly different (*p* > 0.05).

b Mean values (±Standard deviation) in the same column having similar superscripts are not significantly different (*p* > 0.05).

c Mean values (±Standard deviation) in the same column having similar superscripts are not significantly different (*p* > 0.05).

d Mean values (±Standard deviation) in the same column having similar superscripts are not significantly different (*p* > 0.05).

### Testicular cytoarchitectural examination

**FIGURE 11:**
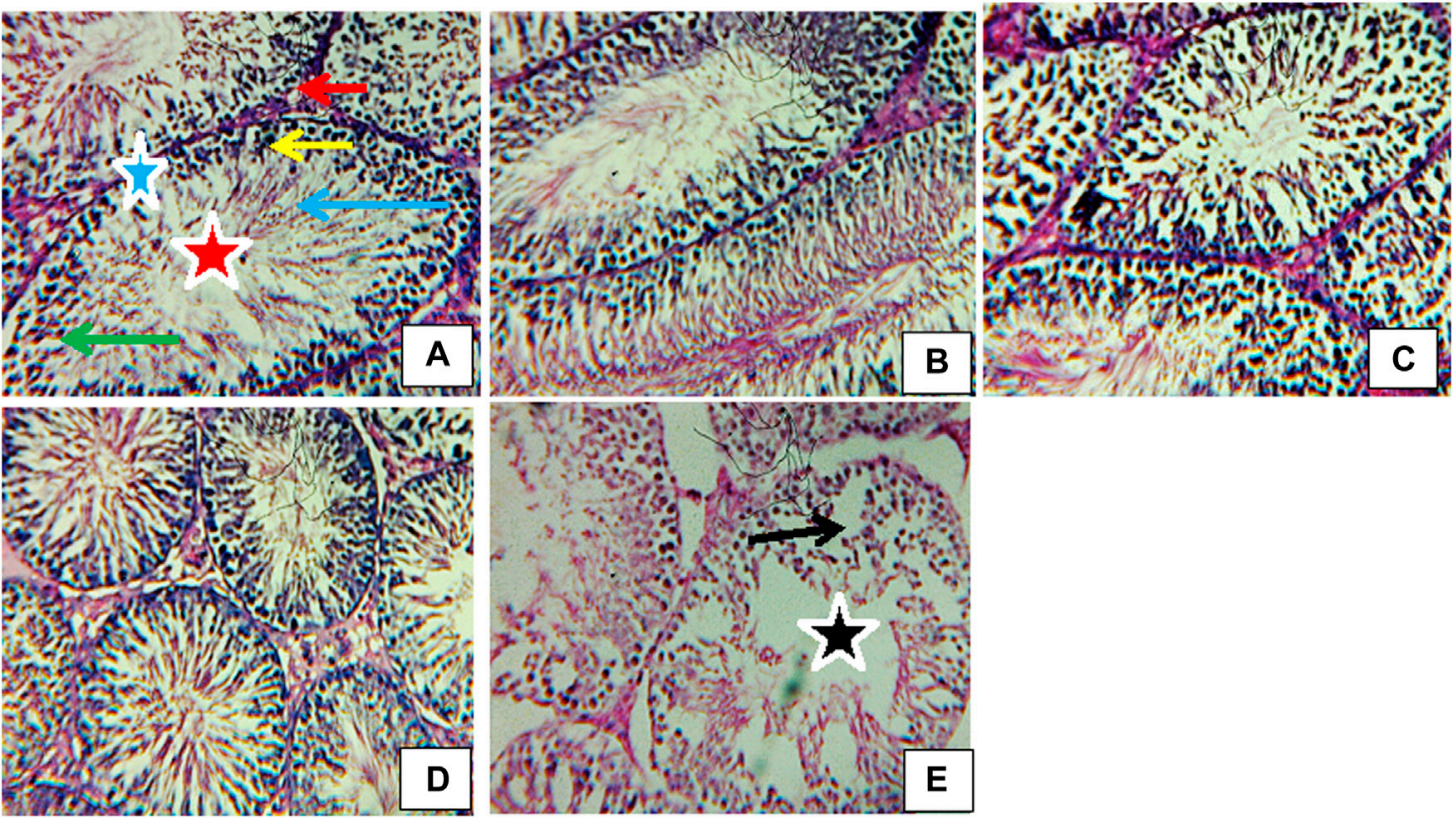
Transverse section of testes (H&E stain; x 400) of **(A)** control revealing normal structure spermatogonia (yellow arrow), spermatids (green arrow), spermatozoa (blue arrow), Leydig cells (red arrow), myofibroblasts (blue star), lumen (red star). **(B)** Rat exposed to atrazine at 0.01 mg/L revealing a normal structure similar to control. **(C)** Rat exposed to atrazine at 0.03 mg/L revealing normal structure similar to control **(D)** rat exposed to atrazine at 0.04 mg/L revealing normal structure similar to control **(E)** rat exposed to atrazine at 0.08 mg/L revealing mild disoriented seminiferous tubule (black star) as well as displacement and depletion of spermatogonia, spermatocytes and spermatids (black arrow) with uneven resting of spermatogonia along the membranes.

Figure 11 displays the photomicrographs of the testes of rats exposed to atrazine concentrations in drinking water from Ijebu-North, Southwest Nigeria. Normal testicular structure, characterized by darkly stained oval and round spermatogonia on the basement membrane with myofibroblast, was observed in control rats and those exposed to atrazine at 0.01, 0.03, and 0.04 mg/L concentrations. In addition, the spermatocytes and spermatids are well seen with a large number of spermatozoa found in the lumen of the seminiferous tubule. The Leydig cells appear normal within the interstitium. Meanwhile, mild disoriented seminiferous tubule, as well as displacement and depletion of spermatogonia, spermatocytes, and spermatids with uneven resting of spermatogonia along the membranes, were observed in the testes structure of rats exposed to atrazine at 0.08 mg/L concentration.

## Discussion

The concentrations of atrazine detected in water from Oru and Ilaporu communities were all below the MAC of 0.005 mg/L established by Canada and 0.003 mg/L established by the United States EPA. Hand-dug well from Ijebu-Igbo contained atrazine concentration up to the MAC of United States EPA. Meanwhile, well waters from Awa and Mamu communities both recorded atrazine concentration that is above the United States EPA limit, but lower than the limit set by Canada. However, the concentration of atrazine above the United States EPA and Canada limits was detected in sampling point 1 of the hand-dug well from the Ago-Iwoye community. This is an indication that hand-dug well water from Awa, Mamu, and Ago-Iwoye communities is polluted with atrazine herbicide. The higher level of atrazine observed in the hand-dug well water from Awa, Mamu, and Ago-Iwoye could be attributed to the possible higher use of atrazine in these farming communities since a high level of atrazine detected in some sites was attributed to the higher use of the herbicide (Sun et al., 2017). Meanwhile, variations and reductions in atrazine concentration detected in water were observed as we moved away from the farm villages of each community. This may be due to the geographical structure, depth of the hand-dug well as well as agricultural activities of the sampled area, which have been reported to influence the spatial distribution of atrazine (Sun et al., 2017; Almasi et al., 2020). The influence of depth on the concentration of atrazine detected in hand-dug well is a possibility. Since the concentration of atrazine in the boreholes was lower than that recorded in the well, it is expected that the depth of boreholes will be greater than that of hand-dug wells.

The HQ values for non-carcinogenic effect through ingestion and dermal routes of exposure for adults and children were less than 1 in all of the community. This indicates that the water from each of the communities may be safe for domestic use. However, the HQ values through the exposure routes for children were higher than that of adults. Our findings confirm Almasi et al. (2020) and Dehghani et al. (2022) who documented higher HQ values for the two exposure routes in adults and children. HQ values for children through dermal contact ranges from 0.001 to 0.030 in Ijebu-Igbo; 0.001 to 0.082 in Ago-Iwoye; 0.001 to 0.010 in Oru; 0.000102 to 0.040 in Awa and 0.001 t0 0.041 in Mamu. While for adults, it ranged from 0.002 to 0.016 in Ijebu-Igbo; 0.001 to 0.045 in Ago-Iwoye; 0.001 to 0.006 in Oru; 0.00056 to 0.023 in Awa and 0.0006 to 0.023 in Mamu. This shows that dermal contact poses a higher risk for children than adults. In addition, Ago-Iwoye and Awa communities are more contaminated and pose the highest risk of water use. But here in this study, we did not evaluate the health risk arising from ingestion of atrazine through food such as corn, as this can also increase the health risk (Sánchez et al., 2019). Thus, our findings in this study should be applied with caution.

The present study shows a significant reduction in the serum level of testosterone hormone only in rats exposed to atrazine at 0.08 mg/L, but no significant difference in the hormone level was observed between control and those exposed to atrazine at 0.01, 0.03, and 0.04 mg/L. Our finding is, in part, supported by Stoker et al. (2000), Stoker et al. (2002) and Abarikwu et al. (2015) who observed no effect on plasma testosterone levels in rats following exposure to atrazine at 12.5 mg/kg. However, Victor-Costa et al. (2010) reported decreased testosterone levels in rats at higher doses of atrazine. A high level of prolactin or hyperprolactinemia observed in rats exposed to atrazine at 0.04 and 0.08 mg/L may possibly explain their reduced testosterone level. Although hyperprolactinemia impairs the proper function of the male gonad by either decreasing the pulse generating activity of gonadotropin-releasing hormone (GnRH) or directly inhibiting the secretion of testosterone at the Leydig cells (Horseman et al., 2006), the insignificant increase in FSH and LH of the exposed rats suggests a dysfunction in the testis of the exposed rats. On the contrary, exposure to atrazine at 200 mg/kg in rats was observed to inhibit GnRH, consequently affecting the synthesis of LH and FSH (Foradori et al., 2013; Wirbisky and Freeman, 2015). Furthermore, a decreased serum LH and FSH in rats exposed to atrazine at 200 and 400 mg/kg has been witnessed (Trentacoste et al., 2001; Mokhtari et al., 2011).

Oxidative stress biomarkers (MDA), the concentration of GSH, and antioxidant enzymes were studied in the testis of rats exposed to atrazine. Exposure to atrazine at concentrations found in water from Ijebu-North induced an increase in MDA level with an increase in atrazine concentration mostly at 0.08 mg/L. This might suggest oxidative stress at this concentration. SOD, an endogenous scavenger that dismutates superoxide radical, was observed to increase at 0.03 and 0.04 mg/L of atrazine exposure but decrease at 0.08 mg/L concentration. The decrease in SOD activity may be due to the inactivation of SOD by interaction with oxygen radicals (Kaushik and Kaur, 2003). Catalase is known to scavenge hydrogen peroxide at higher concentrations (Alptekin et al., 1996) and at lower concentrations by GPx (Cohen and Hochstein, 1963). In this study, the relative contributions of CAT and GPx in the decomposition of hydrogen peroxide are concentration-specific with CAT being more potent at 0.01, 0.03, and 0.04 mg/L of atrazine concentration but at 0.08 mg/L, while GPx is being impotent at the increasing concentration of atrazine. An increased CAT activity observed in the testes of atrazine-exposed rats signifies that the enzyme is more responsive to the increased hydrogen peroxide concentration in the testes tissue, therefore, emphasizing its role in the control of cellular lipid peroxide concentration (Spasic´ et al., 1993). This is very important and significant, especially in the maintenance of cellular proliferation and differentiation in the testes. The decreased activity of GPx in the testes of the atrazine-exposed rats indicates its inability or reduced capacity to scavenge hydrogen peroxide generated in the tissue in response to atrazine exposure. Our finding is, in part, supported by Singh et al. (2010) and Qian et al. (2008) who found increased activities of CAT and SOD as a compensatory or adaptive mechanism in response to oxidative stress induced by atrazine. This claim is reflected in the insignificant alteration seen in the testicular MDA level of the atrazine-exposed rats at 0.01, 0.03, and 0.04 mg/L, which enables us to suggest that the antioxidant defence system in the testes protects the organ from ROS induced by the herbicide. Meanwhile, our finding partially contradicts Abarikwu et al. (2014) who reported a decreased SOD activity, unchanged GSH concentration and increased MDA level in the testes of rats exposed to atrazine at 120 mg/kg, but CAT activity was unaffected (Abarikwu et al., 2010).

GST is one of the primary detoxification enzymes and a rise in the activity of this enzyme may therefore indicate its ability to rapidly detoxify environmental xenobiotics. In addition, a rise in the GST activity observed in the testis of rats in response to atrazine exposure is accompanied by a significant reduction in its GSH concentration suggesting a rise in conjugation reactions. While Kaushik and Kaur (2003)) suggested that a decreased GSH concentration may directly result from a reduction in its synthesis or an increase in its breakdown during stress, Simmons et al. (1990) and Alptekin et al. (1996) attributed a decreased GSH concentration to increased lipid peroxidation. But here in this study, increased consumption of GSH in conjugation reactions due to increased GST seems to explain the GSH concentration reductions in the testis of atrazine-exposed rats. This is understandable since the detoxification of pesticides is mainly through a reaction catalyzed by GST, which utilizes GSH. On the contrary, a decreased GST activity and unchanged GSH concentration in the testes of rats exposed to 120 and 200 mg/kg atrazine after 16 days has been reported (Abarikwu et al., 2010).

As reported by Kempaiah and Srinivasan (Kempaiah and Srinivasan, 2006), membrane-bound ATPase enzymes are good markers of membrane dysfunctions under oxidative stress. The enzymes supply energy via hydrolytic reaction to control membrane permeability and transportation of ions such as Ca2+, K+, and Na + across the membrane (Langeswaran et al., 2012). We showed for the first time in the present study that exposure to atrazine at 0.01, 0.03, 0.04, and 0.08 mg/L has no significant effect on the activities of membrane-bound ATPases in the testes of exposed rats. This suggests that atrazine at those concentrations may not disturb the ion homeostasis of the testes or alter cellular metabolism. Atrazine at those concentrations may not also change cell membrane integrity and permeability or induce a rise in membrane fluidity and disturbances in vital functions (Chodon et al., 2008).

A significant reduction in percentage sperm motility was observed in the experimental rats with an increase in the concentration of atrazine exposure. However, sperm count was relatively unaffected in the experimental rats. Our finding is, in part, supported by Kniewald et al. (2000) and Abarikwu et al. (2014) who observed a decrease in sperm count and motility in rats exposed to atrazine. Atrazine seems to target the sperm tail of the experimental rats since the number of sperms with the abnormal tail was observed to significantly contribute to the total abnormal sperm of the experimental rats. This may possibly explain the low motility observed in the sperm. Although the mechanism underlying the tail abnormality by atrazine is unknown in the present study, a further mechanism-based approach study focusing on this area is hereby recommended. However, atrazine did not induce significant sperm head abnormalities in this present study. Our findings on sperm morphology agree with Abarikwu et al. (2014) who reported insignificant head abnormalities of sperms, but higher sperm tail and midpiece abnormalities in rats exposed to atrazine at 120 mg/kg.

The observed mild changes in the testicular cytoarchitectural structure of rats exposed to atrazine at 0.08 mg/L may indicate mild toxicity to the male reproductive system of the rats, which may consequently interfere with the spermatogenesis process (Thakur et al., 2014). Although a concentration-dependent increase in sperm motility and morphology was observed in rats exposed to atrazine at 0.01, 0.03, and 0.04 mg/L concentrations, unlike atrazine at 0.08 mg/L, the testicular antioxidant defence was strong enough to protect the structural integrity of the testes of rats exposed to these concentrations.

## Conclusion

Our findings show the presence of atrazine residue in HDW, boreholes, and stream waters from the communities in Ijebu-North LGA, southwest Nigeria. The HI values for non-carcinogenic effect via ingestion and dermal routes for children and adults were below the acceptable limit for all the communities. But children from the LGA seem to be more at risk than adults. In addition, Ago-Iwoye and Awa communities were more contaminated and may pose the highest risk of water use. Atrazine at environmentally relevant concentrations of 0.01, 0.03, and 0.04 mg/L was observed to induce testicular antioxidant defence capable of protecting the tissue against oxidative damage, degenerative lesions, and reproductive dysfunction. However, atrazine at 0.08 mg/L may induce mild reproductive toxicity in the exposed animal. As part of the requirements of FQPA on water quality assessment, this is the first study to monitor the level of atrazine in water from HDW, boreholes, and streams of rural areas in Nigeria and could contribute to the establishment of MCL of atrazine in drinking water in Nigeria. This study could also contribute to the ongoing USDA Pesticide Data Program monitoring for pesticide residues in food and water. We recommend prompt intervention by the regulatory agencies and policymakers on the use of atrazine herbicide in rural agricultural areas of Ijebu-North southwest, Nigeria. In addition, there is a need to further analyse the testis of the exposed animal for any evidence of atrazine accumulation. Since the exposure to atrazine concentrations for 12 weeks (sub-chronic toxicity study) in the present study was observed to induce little or no effect on the testis, there is a need for chronic exposure to the same atrazine concentrations in the future study.

## Data Availability

The raw data supporting the conclusion of this article will be made available by the authors, without undue reservation.
