# Supplementary material for: First report on atrazine monitoring in drinking water from Ijebu-North, South-West Nigeria: Human health risk evaluation and reproductive toxicity studies
Source: Front Toxicol. 2022 Sep 26;4:975636. doi: 10.3389/ftox.2022.975636 (PMC9549142; doi:10.3389/ftox.2022.975636)
Supplement: Supplementary file 1 [file DataSheet1.docx]

Supplementary Material

Supplementary Table 1: Number of water sample collected from each of the communities.

| Community | Number of HDW | Number of BH | Number of stream |
| --- | --- | --- | --- |
| Ago-iwoye | 20 | 7 | 1 |
| Oru | 9 | 7 | I |
| Ilaporu | 10 | None | None |
| Awa | 7 | 5 | None |
| Ijebu-igbo | 10 | 10 | None |
| Mamu | 3 | 1 | 2 |
| Abeokuta | 10 | 10 | None |

HDW-hand dug well; BH- borehole

Supplementary Table 2a; Depth of hand dug well and coordinates of sampling locations n Awa rural community

| **WELL SAPLING POINT** | **WELL DEPTH (m)** | **BOREHOLE SAPLING POINT** | **Latitude** | **Longitude** | **Latitude** | **Longitude** |
| --- | --- | --- | --- | --- | --- | --- |
|  |  | 1 | 6⁰ 57' 11ʺ | 3⁰ 55' 50ʺ | 6.954385 | 3.931431 |
| 1 | 9.3 |  | 6⁰ 57' 19ʺ | 3⁰ 55' 59ʺ | 6.955716 | 3.933162 |
|  |  | 2 | 6⁰ 57' 19ʺ | 3⁰ 56' 12ʺ | 6.955443 | 3.936687 |
| 2 | 10.7 |  | 6⁰ 57' 30ʺ | 3⁰ 56' 03ʺ | 6.958566 | 3.934287 |
|  |  | 3 | 6⁰ 57' 30ʺ | 3⁰ 56' 07ʺ | 6.958564 | 3.935531 |
| 3 | 10.5 |  | 6⁰ 57' 29ʺ | 3⁰ 56' 08ʺ | 6.958302 | 3.935626 |
|  |  | 4 | 6⁰ 57' 38ʺ | 3⁰ 56' 08ʺ | 6.960577 | 3.935799 |
| 4 | 9.6 |  | 6⁰ 57' 38ʺ | 3⁰ 56' 12ʺ | 6.960412 | 3.937006 |
| 5 | 9.8 |  | 6⁰ 57' 32ʺ | 3⁰ 56' 17ʺ | 6.959022 | 3.938106 |
| 6 | 11.3 |  | 6⁰ 57' 32ʺ | 3⁰ 56' 13ʺ | 6.959002 | 3.937064 |
| 7 | 11.2 |  | 6⁰ 57' 34ʺ | 3⁰ 56' 15ʺ | 6.959384 | 3.937533 |
|  |  | 5 | 6⁰ 57' 47ʺ | 3⁰ 56' 16ʺ | 6.959384 | 3.937924 |

Supplementary Table 2b; Depth of hand dug well and coordinates of sampling locations n Ago-Iwoye rural community

| **WELL SAPLING POINT** | **WELL DEPTH**  **(m)** | **BOREHOLE SAPLING POINT** | **STREAM SAPLING POINT** | **Latitude** | **Longitude** | **Latitude** | **Longitude** |
| --- | --- | --- | --- | --- | --- | --- | --- |
|  |  | 1 |  | 6⁰ 57' 26ʺ | 3⁰ 47' 33ʺ | 6.957213 | 3.792494 |
|  |  |  | 1 | 6⁰ 58' 00ʺ | 3⁰ 47' 48ʺ | 6.966891 | 3.796848 |
| 1 | 9.4 |  |  | 6⁰ 57' 42ʺ | 3⁰ 47' 44ʺ | 6.961764 | 3.71511 |
|  |  | 2 |  | 6⁰ 58' 13ʺ | 3⁰ 48' 55ʺ | 6.970438 | 3.815324 |
| 2 | 9.6 |  |  | 6⁰ 58' 13ʺ | 3⁰ 48' 56ʺ | 6.970307 | 3.815587 |
|  |  | 3 |  | 6⁰ 56' 28ʺ | 3⁰ 49' 49ʺ | 6.941275 | 3.830293 |
| 3 | 8.2 |  |  | 6⁰ 56' 30ʺ | 3⁰ 49' 47ʺ | 6.941926 | 3.829887 |
| 4 | 10.7 |  |  | 6⁰ 56' 36ʺ | 3⁰ 51' 23ʺ | 6.943326 | 3.856437 |
| 5 | 10.3 |  |  | 6⁰ 56' 05ʺ | 3⁰ 54' 02ʺ | 6.93426 | 3.899488 |
| 6 | 7.9 |  |  | 6⁰ 56' 14ʺ | 3⁰ 55' 40ʺ | 6.937455 | 3.927837 |
|  |  | 4 |  | 6⁰ 56' 14ʺ | 3⁰ 54' 32ʺ | 6.935478 | 3.901958 |
| 7 | 9.3 |  |  | 6⁰ 54' 54ʺ | 3⁰ 54' 70ʺ | 6.948513 | 3.911295 |
| 8 | 8.5 |  |  | 6⁰ 57' 19ʺ | 3⁰ 54' 34ʺ | 6.955423 | 3.9095 |
|  |  | 5 |  | 6⁰ 56' 53ʺ | 3⁰ 34' 47ʺ | 6.94812 | 3.913214 |
| 9 | 7.3 |  |  | 6⁰ 56' 35ʺ | 3⁰ 56' 10ʺ | 6.943198 | 3.936112 |
| 10 | 6.8 |  |  | 6⁰ 56' 39ʺ | 3⁰ 54' 06ʺ | 6.944383 | 3.901814 |
| 11 | 8.1 |  |  | 6⁰ 96' 43ʺ | 3⁰ 56' 00ʺ | 6.945527 | 3.933421 |
| 12 | 7.4 |  |  | 6⁰ 56' 29ʺ | 3⁰ 55' 24ʺ | 6.944169 | 3.923396 |
|  |  | 6 |  | 6⁰ 56' 36ʺ | 3⁰ 55' 27ʺ | 6.943538 | 3.924313 |
| 13 | 9.2 |  |  | 6⁰ 56' 07ʺ | 3⁰ 54' 51ʺ | 6.935404 | 3.914248 |
| 14 | 7.6 |  |  | 6⁰ 56' 06ʺ | 3⁰ 54' 37ʺ | 6.935077 | 3.910467 |
| 15 | 6.4 |  |  | 6⁰ 56' 18ʺ | 3⁰ 54' 55ʺ | 6.938366 | 3.915509 |
| 16 | 9.2 |  |  | 6⁰ 55' 50ʺ | 3⁰ 55' 00ʺ | 6.930653 | 3.911678 |
| 17 | 11.5 |  |  | 6⁰ 56' 32ʺ | 3⁰ 55' 19ʺ | 6.942404 | 3.921963 |
| 18 | 10.4 |  |  | 6⁰ 56' 31ʺ | 3⁰ 55' 06ʺ | 6.941937 | 3.918583 |
|  |  | 7 |  | 6⁰ 56' 33ʺ | 3⁰ 55' 12ʺ | 6.941992 | 3.920113 |
| 19 | 7.7 |  |  | 6⁰ 56' 20ʺ | 3⁰ 55' 27ʺ | 6.938983 | 3.924218 |
| 20 | 8.3 |  |  | 6⁰ 56' 07ʺ | 3⁰ 55' 37ʺ | 6.935277 | 3.927134 |

Supplementary Table 2c; Depth of hand dug well and coordinates of sampling locations n Mamu rural community

| **WELL SAPLING POINT** | **WELL DEPTH (m)** | **BOREHOLE SAPLING POINT** | **STREAM SAPLING POINT** | **Latitude** | **Longitude** | **Latitude** | **Longitude** |
| --- | --- | --- | --- | --- | --- | --- | --- |
| 1 | 10.8 |  |  | 7⁰ 04' 54ʺ | 3⁰ 53' 45ʺ | 7.087111 | 3.9105 |
| 2 | 10.3 |  |  | 7⁰ 04' 19ʺ | 3⁰ 54' 10ʺ | 7.0722 | 3.903 |
|  |  |  | 1 | 7⁰ 05' 19ʺ | 3⁰ 54' 27ʺ | 7.0888 | 3.9077 |
|  |  | 1 |  | 7⁰ 05' 00ʺ | 3⁰ 54' 40ʺ | 7.083571 | 3.911181 |
| 3 | 11.6 |  |  | 7⁰ 04' 56ʺ | 3⁰ 54' 36ʺ | 7.082308 | 3.91004 |
|  |  |  | 2 | 7⁰ 42' 23ʺ | 3⁰ 54' 34ʺ | 7.081191 | 3.909645 |

Supplementary Table 2d; Depth of hand dug well and coordinates of sampling locations n Oru rural community

| **WELL SAPLING POINT** | **WELL DEPTH (m)** | **BOREHOLE SAPLING POINT** | **STREAM SAPLING POINT** | **Latitude** | **Longitude** | **Latitude** | **Longitude** |
| --- | --- | --- | --- | --- | --- | --- | --- |
|  |  | 1 |  | 6⁰ 57' 08ʺ | 3⁰ 56' 18ʺ | 6.95236 | 3.93856 |
| 1 | 7.4 |  |  | 6⁰ 57' 11ʺ | 3⁰ 56' 24ʺ | 6.95314 | 3.94014 |
|  |  | 2 |  | 6⁰ 56' 51ʺ | 3⁰ 56' 30ʺ | 6.94752 | 3.94178 |
| 2 | 6.5 |  |  | 6⁰ 56' 51ʺ | 3⁰ 56' 31ʺ | 6.94754 | 3.94193 |
|  |  | 3 |  | 6⁰ 56' 47ʺ | 3⁰ 56' 40ʺ | 6.9466 | 3.94469 |
| 3 | 6.2 |  |  | 6⁰ 56' 53ʺ | 3⁰ 56' 39ʺ | 6.94813 | 3.94431 |
|  |  | 4 |  | 6⁰ 56' 34ʺ | 3⁰ 56' 18ʺ | 6.94301 | 3.94656 |
| 4 | 8.3 |  |  | 6⁰ 56' 35ʺ | 3⁰ 56' 48ʺ | 6.94312 | 3.9468 |
|  |  |  | 1 | 6⁰ 56' 46ʺ | 3⁰ 57' 01ʺ | 6.94633 | 3.95038 |
| 5 | 7.1 |  |  | 6⁰ 56' 46ʺ | 3⁰ 56' 56ʺ | 6.94619 | 3.94888 |
| 6 | 9.4 |  |  | 6⁰ 56' 53ʺ | 3⁰ 56' 56ʺ | 6.94817 | 3.94907 |
| 7 | 8.8 |  |  | 6⁰ 57' 07ʺ | 3⁰ 57' 02ʺ | 6.59199 | 3.95078 |
|  |  | 5 |  | 6⁰ 57' 11ʺ | 3⁰ 56' 51ʺ | 6.95316 | 3.94756 |
| 8 | 6.9 |  |  | 6⁰ 57' 15ʺ | 3⁰ 56' 31ʺ | 6.95431 | 3.94201 |
|  |  | 6 |  | 6⁰ 57' 15ʺ | 3⁰ 56' 26ʺ | 6.95437 | 3.94065 |
|  |  | 7 |  | 6⁰ 57' 01ʺ | 3⁰ 56' 36ʺ | 6.95032 | 3.94359 |
| 9 | 7.2 |  |  | 6⁰ 57' 09ʺ | 3⁰ 56' 30ʺ | 6.95276 | 3.94174 |

Supplementary Table 2e; Depth of hand dug well and coordinates of sampling locations n Ilaporu rural community

| **WELL SAPLING POINT** | **WELL DEPTH (m)** | **Latitude** | **Longitude** | **Latitude** | **Longitude** |
| --- | --- | --- | --- | --- | --- |
| 1 | 9.2 | 6⁰ 57' 32ʺ | 3⁰ 56' 30ʺ | 6.959 | 3.9418 |
| 2 | 10.6 | 6⁰ 57' 30ʺ | 3⁰ 56' 26ʺ | 6.9585 | 3.9406 |
| 3 | 8.4 | 6⁰ 57' 30ʺ | 3⁰ 56' 36ʺ | 6.95853 | 3.94337 |
| 4 | 7.9 | 6⁰ 57' 22ʺ | 3⁰ 56' 24ʺ | 6.956198 | 3.940163 |
| 5 | 9.1 | 6⁰ 57' 33ʺ | 3⁰ 56' 40ʺ | 6.959419 | 3.944441 |
| 6 | 10.4 | 6⁰ 57' 32ʺ | 3⁰ 56' 38ʺ | 6.95915 | 3.94408 |
| 7 | 9.5 | 6⁰ 57' 37ʺ | 3⁰ 56' 43ʺ | 6.960386 | 3.945357 |
| 8 | 9.3 | 6⁰ 57' 34ʺ | 3⁰ 56' 44ʺ | 6.959462 | 3.945719 |
| 9 | 10.3 | 6⁰ 57' 35ʺ | 3⁰ 56' 32ʺ | 6.959842 | 3.942421 |
| 10 | 9.4 | 6⁰ 57' 41ʺ | 3⁰ 56' 37ʺ | 6.961625 | 3.943643 |

Supplementary Table 2f; Depth of hand dug well and coordinates of sampling locations n Ijebu-Igbo rural community

| **WELL SAPLING POINT** | **WELL DEPTH (m)** | **BOREHOLE SAPLING POINT** | **Latitude** | **Longitude** | **Latitude** | **Longitude** |
| --- | --- | --- | --- | --- | --- | --- |
|  |  | 1 | 6⁰ 57' 31ʺ | 3⁰ 59' 26ʺ | 6.958847 | 3.984057 |
| 1 | 9.4 |  | 6⁰ 57' 31ʺ | 3⁰ 59' 28ʺ | 6.958806 | 3.984173 |
|  |  | 2 | 6⁰ 57' 91ʺ | 3⁰ 59' 73ʺ | 6.9653 | 3.9955 |
| 2 | 8.7 |  | 6⁰ 57' 90ʺ | 3⁰ 59' 70ʺ | 6.9657 | 3.9946 |
|  |  | 3 | 6⁰ 36' 14ʺ | 3⁰ 38' 18ʺ | 6.603936 | 3.638397 |
| 3 | 9.6 |  | 6⁰ 57' 55ʺ | 4⁰ 00' 21ʺ | 6.9593 | 4.0036 |
|  |  | 4 | 6⁰ 57' 27ʺ | 4⁰ 00' 58ʺ | 6.9491 | 4.0104 |
| 4 | 8.8 |  | 6⁰ 57' 35ʺ | 4⁰ 00' 64ʺ | 6.949 | 4.0097 |
|  |  | 5 | 6⁰ 59' 07ʺ | 3⁰ 59' 35ʺ | 6.983551 | 3.993185 |
| 5 | 8.1 |  | 6⁰ 59' 42ʺ | 3⁰ 59' 38ʺ | 6.984493 | 3.994046 |
|  |  | 6 | 6⁰ 58' 49ʺ | 3⁰ 59' 40ʺ | 6.980382 | 3.994592 |
| 6 | 8.5 |  | 6⁰ 58' 49ʺ | 3⁰ 59' 40ʺ | 6.980382 | 3.994592 |
|  |  | 7 | 6⁰ 58' 26ʺ | 3⁰ 59' 22ʺ | 6.974059 | 3.98964 |
| 7 | 9.4 |  | 6⁰ 58' 24ʺ | 3⁰ 59' 23ʺ | 6.97349 | 3.989811 |
|  |  | 8 | 6⁰ 58' 41ʺ | 3⁰ 59' 24ʺ | 6.978184 | 3.99013 |
| 8 | 8.7 |  | 6⁰ 58' 41ʺ | 3⁰ 59' 25ʺ | 6.977893 | 3.990309 |
|  |  | 9 | 6⁰ 59' 13ʺ | 4⁰ 04' 39ʺ | 6.986949 | 4.01222 |
| 9 | 10.5 |  | 6⁰ 59' 12ʺ | 4⁰ 04' 36ʺ | 6.98687 | 4.012126 |
|  |  | 10 | 6⁰ 58' 57ʺ | 4⁰ 01' 90ʺ | 6.982696 | 4.005335 |
| 10 |  |  | 6⁰ 58' 56ʺ | 4⁰ 01' 71ʺ | 6.982307 | 4.004773 |

Supplementary Table 2g; Depth of hand dug well and coordinates of sampling locations n Abeokuta urban area

| **WELL SAPLING POINT** | **WELL DEPTH (m)** | **BOREHOLE SAPLING POINT** | **Latitude** | **Longitude** | **Latitude** | **Longitude** |
| --- | --- | --- | --- | --- | --- | --- |
| 1 | 9.2 |  | 7⁰ 07' 10ʺ | 3⁰ 22' 58ʺ | 7.119492 | 3.382908 |
| 2 | 8.6 |  | 7⁰ 07' 02ʺ | 3⁰ 23' 11ʺ | 7.117398 | 3.386417 |
| 3 | 8.0 |  | 7⁰ 07' 45ʺ | 3⁰ 22' 32ʺ | 7.129397 | 3.375693 |
| 4 | 8.4 |  | 7⁰ 08' 09ʺ | 3⁰ 20' 13ʺ | 7.135875 | 3.33718 |
|  |  | 1 | 7⁰ 08' 10ʺ | 3⁰ 20' 12ʺ | 7.136242 | 3.336659 |
| 5 | 9.1 |  | 7⁰ 07' 46ʺ | 3⁰ 20' 02ʺ | 7.127968 | 3.33406 |
|  |  | 2 | 7⁰ 07' 37ʺ | 3⁰ 19' 58ʺ | 7.127029 | 3.332898 |
|  |  | 3 | 7⁰ 07' 44ʺ | 3⁰ 20' 04ʺ | 7.128979 | 3.334454 |
| 6 | 9.9 |  | 7⁰ 07' 35ʺ | 3⁰ 20' 24ʺ | 7.126486 | 3.340136 |
|  |  | 4 | 7⁰ 07' 35ʺ | 3⁰ 20' 26ʺ | 7.126486 | 3.340136 |
|  |  | 5 | 7⁰ 07' 35ʺ | 3⁰ 20' 25ʺ | 7.12638 | 3.340066 |
|  |  | 6 | 7⁰ 07' 43ʺ | 3⁰ 21' 00ʺ | 7.128686 | 3.349999 |
|  |  | 7 | 7⁰ 07' 34ʺ | 3⁰ 20' 03ʺ | 7.126202 | 3.339922 |
| 7 | 8.6 |  | 7⁰ 08' 13ʺ | 3⁰ 21' 58ʺ | 7.136943 | 3.366149 |
|  |  | 8 | 7⁰ 09' 40ʺ | 3⁰ 22' 31ʺ | 7.16126 | 3.375325 |
|  |  | 9 | 7⁰ 10' 17ʺ | 3⁰ 23' 02ʺ | 7.171567 | 3.384094 |
| 8 | 10.1 |  | 7⁰ 09' 53ʺ | 3⁰ 22' 42ʺ | 7.164904 | 3.378432 |
| 9 | 9.5 |  | 7⁰ 11' 06ʺ | 3⁰ 22' 26ʺ | 7.185195 | 3.373952 |
| 10 | 8.6 |  | 7⁰ 10' 56ʺ | 3⁰ 22' 16ʺ | 7.182313 | 3.37135 |
|  |  | 10 | 7⁰ 07' 36ʺ | 3⁰ 20' 03ʺ | 7.126843 | 3.334199 |

Supplementary Table 3a: Hazard quotient for adults and children associated with exposure to atrazine concentration in hand dug well water from Ijebu-Igbo community

|  | | **INGESTION** | | | | **DERMAL** | | | |
| --- | --- | --- | --- | --- | --- | --- | --- | --- | --- |
|  |  | **ADULT** | | **CHILDREN** | | **ADULT** |  | **CHILDREN** | |
| **SP** | **CON** | **CDI** | **HQ** | **CDI** | **HQ** | **CDI** | **HQ** | **CDI** | **HQ** |
| 1 | 0.03 | $8.21\times{10}^{-4}$ | 0.023 | $1.91\times{10}^{-3}$ | 0.054 | $5.91\times{10}^{-4}$ | 0.016 | $1.07\times{10}^{-3}$ | 0.030 |
| 2 | 0.03 | $8.21\times{10}^{-4}$ | 0.023 | $1.91\times{10}^{-3}$ | 0.054 | $5.91\times{10}^{-4}$ | 0.016 | $1.07\times{10}^{-3}$ | 0.030 |
| 3 | ND | $ND$ | ND | ND | ND | ND | ND | ND | ND |
| 4 | 0.01 | $2.73\times{10}^{-4}$ | 0.007 | $6.39\times{10}^{-4}$ | 0.018 | $1.97\times{10}^{-4}$ | 0.005 | $3.57\times{10}^{-4}$ | 0.01 |
| 5 | 0.02 | $5.47\times{10}^{-4}$ | 0.015 | $1.27\times{10}^{-3}$ | 0.036 | $3.94\times{10}^{-4}$ | 0.011 | $7.15\times{10}^{-4}$ | 0.020 |
| 6 | ND | ND | ND | ND | ND | ND | ND | ND | ND |
| 7 | ND | ND | ND | ND | ND | ND | ND | ND | ND |
| 8 | ND | ND | ND | ND | ND | ND | ND | ND | ND |
| 9 | 0.02 | $5.47\times{10}^{-4}$ | 0.015 | $1.27\times{10}^{-3}$ | 0.036 | $3.94\times{10}^{-4}$ | 0.011 | $7.15\times{10}^{-4}$ | 0.020 |
| 10 | ND | ND | ND | ND | ND | ND | ND | ND | ND |

Supplementary Table 3b: Hazard quotient for adults and children associated with exposure to atrazine concentration in borehole water from Ijebu-Igbo community

|  | | **INGESTION** | | | | | **DERMAL** | | | | |
| --- | --- | --- | --- | --- | --- | --- | --- | --- | --- | --- | --- |
|  |  | **ADULT** | | **CHILDREN** | | | **ADULT** |  | **CHILDREN** | | |
| **SP** | **CON** | **CDI** | **HQ** | **CDI** | | **HQ** | **CDI** | **HQ** | **CDI** | | **HQ** |
| 1 | 0.01 | $2.73\times{10}^{-4}$ | 0.007 | | $6.39\times{10}^{-4}$ | 0.018 | $1.97\times{10}^{-4}$ | 0.005 | | $3.57\times{10}^{-4}$ | 0.010 |
| 2 | 0.003 | $8.21\times{10}^{-5}$ | 0.002 | | $1.91\times{10}^{-4}$ | 0.005 | $5.91\times{10}^{-5}$ | 0.002 | | $1.07\times{10}^{-4}$ | 0.003 |
| 3 | ND | ND | ND | | ND | ND | ND | ND | | ND | ND |
| 4 | ND | ND | ND | | ND | ND | ND | ND | | ND | ND |
| 5 | 0.003 | $8.21\times{10}^{-5}$ | 0.002 | | $1.91\times{10}^{-4}$ | 0.005 | $5.91\times{10}^{-5}$ | 0.002 | | $1.07\times{10}^{-4}$ | 0.003 |
| 6 | ND | ND | ND | | ND | ND | ND | ND | | ND | ND |
| 7 | BDL | NIL | NIL | | NIL | NIL | NIL | NIL | | NIL | NIL |
| 8 | ND | ND | ND | | ND | ND | ND | ND | | ND | ND |
| 9 | 0.003 | $8.21\times{10}^{-5}$ | 0.002 | | $1.91\times{10}^{-4}$ | 0.005 | $5.91\times{10}^{-5}$ | 0.002 | | $1.07\times{10}^{-4}$ | 0.003 |
| 10 | ND | ND | ND | | ND | ND | ND | ND | | ND | ND |

Supplementary Table 4a: Hazard quotient for adults and children associated with exposure to atrazine concentration in hand dug well water from Ago-Iwoye community

|  | | **INGESTION** | | | | **DERMAL** | | | |
| --- | --- | --- | --- | --- | --- | --- | --- | --- | --- |
|  |  | **ADULT** | | **CHILDREN** | | **ADULT** |  | **CHILDREN** | |
| **SP** | **CON** | **CDI** | **HQ** | **CDI** | **HQ** | **CDI** | **HQ** | **CDI** | **HQ** |
| 1 | 0.08 | $2.19\times{10}^{-3}$ | 0.063 | $5.11\times{10}^{-3}$ | 0.146 | $1.57\times{10}^{-3}$ | 0.045 | $2.86\times{10}^{-3}$ | 0.082 |
| 2 | 0.03 | $8.21\times{10}^{-4}$ | 0.023 | $1.91\times{10}^{-3}$ | 0.054 | $5.91\times{10}^{-4}$ | 0.016 | $1.07\times{10}^{-3}$ | 0.030 |
| 3 | 0.02 | $5.47\times{10}^{-4}$ | 0.015 | $1.27\times{10}^{-3}$ | 0.036 | $3.94\times{10}^{-4}$ | 0.011 | $7.15\times{10}^{-3}$ | 0.020 |
| 4 | 0.03 | $8.21\times{10}^{-4}$ | 0.023 | $1.91\times{10}^{-3}$ | 0.054 | $5.91\times{10}^{-4}$ | 0.016 | $1.07\times{10}^{-3}$ | 0.030 |
| 5 | 0.02 | $5.47\times{10}^{-4}$ | 0.015 | $1.27\times{10}^{-3}$ | 0.036 | $3.94\times{10}^{-4}$ | 0.011 | $7.15\times{10}^{-3}$ | 0.020 |
| 6 | 0.02 | $5.47\times{10}^{-4}$ | 0.015 | $1.27\times{10}^{-3}$ | 0.036 | $3.94\times{10}^{-4}$ | 0.011 | $7.15\times{10}^{-3}$ | 0.020 |
| 7 | 0.01 | $2.74\times{10}^{-4}$ | 0.007 | $6.39\times{10}^{-4}$ | 0.018 | $1.97\times{10}^{-4}$ | 0.005 | $3.57\times{10}^{-4}$ | 0.010 |
| 8 | 0.02 | $5.47\times{10}^{-4}$ | 0.015 | $1.27\times{10}^{-3}$ | 0.036 | $3.94\times{10}^{-4}$ | 0.011 | $7.15\times{10}^{-3}$ | 0.020 |
| 9 | ND | ND | ND | ND | ND | ND | ND | ND | ND |
| 10 | 0.02 | $5.47\times{10}^{-4}$ | 0.015 | $1.27\times{10}^{-3}$ | 0.036 | $3.94\times{10}^{-4}$ | 0.011 | $7.15\times{10}^{-3}$ | 0.020 |
| 11 | ND | ND | ND | ND | ND | ND | ND | ND | ND |
| 12 | 0.01 | $2.74\times{10}^{-4}$ | 0.008 | $6.39\times{10}^{-4}$ | 0.018 | $1.97\times{10}^{-4}$ | 0.005 | $3.57\times{10}^{-4}$ | 0.010 |
| 13 | 0.01 | $2.74\times{10}^{-4}$ | 0.008 | $6.39\times{10}^{-4}$ | 0.018 | $1.97\times{10}^{-4}$ | 0.005 | $3.57\times{10}^{-4}$ | 0.010 |
| 14 | ND | ND | ND | ND | ND | ND | ND | ND | ND |
| 15 | 0.01 | $2.74\times{10}^{-4}$ | 0.008 | $6.39\times{10}^{-4}$ | 0.018 | $1.97\times{10}^{-4}$ | 0.005 | $3.57\times{10}^{-4}$ | 0.010 |
| 16 | 0.004 | $1.09\times{10}^{-4}$ | 0.003 | $2..55\times{10}^{-4}$ | 0.073 | $7.89\times{10}^{-5}$ | 0.002 | $1.43\times{10}^{-4}$ | 0.004 |
| 17 | ND | ND | ND | ND | ND | ND | ND | ND | ND |
| 18 | 0.003 | $8.22\times{10}^{-5}$ | 0.002 | $1.92\times{10}^{-3}$ | 0.055 | $5.91\times{10}^{-4}$ | 0.002 | $1.07\times{10}^{-4}$ | 0.003 |
| 19 | ND | ND | ND | ND | ND | ND | ND | ND | ND |
| 20 | ND | ND | ND | ND | ND | ND | ND | ND | ND |

Supplementary Table 4b: Hazard quotient for adults and children associated with exposure to atrazine concentration in borehole water from Ago-Iwoye community

|  |  | **INGESTION** | | | | **DERMAL** | | | |
| --- | --- | --- | --- | --- | --- | --- | --- | --- | --- |
| **SP** |  | **ADULT** | | **CHILDREN** | | **ADULT** | | **CHILDREN** | |
|  | **CON** | **CDI** | **HQ** | **CDI** | **HQ** | **CDI** | **HQ** | **CDI** | **HQ** |
| 1 | 0.01 | $2.73\times{10}^{-4}$ | 0.078 | $6.39\times{10}^{-4}$ | 0.018 | $1.97\times{10}^{-4}$ | 0.005 | $3.57\times{10}^{-4}$ | 0.010 |
| 2 | BDL | NIL | NIL | NIL | NIL | NIL | NIL | NIL | NIL |
| 3 | 0.003 | $8.21\times{10}^{-5}$ | 0.002 | $1.91\times{10}^{-4}$ | 0.005 | $5.91\times{10}^{-4}$ | 0.001 | $1.07\times{10}^{-4}$ | 0.003 |
| 4 | BDL | NIL | NIL | NIL | NIL | NIL | NIL | NIL | NIL |
| 5 | BDL | NIL | NIL | NIL | NIL | NIL | NIL | NIL | NIL |
| 6 | 0.003 | $8.21\times{10}^{-5}$ | 0.002 | $1.91\times{10}^{-4}$ | 0.005 | $5.91\times{10}^{-4}$ | 0.001 | $1.07\times{10}^{-4}$ | 0.003 |
| 7 | BDL | NIL | NIL | NIL | NIL | NIL | NIL | NIL | NIL |

Supplementary Table 4c: Hazard quotient for adults and children associated with exposure to atrazine concentration in stream water from Ago-Iwoye community

|  |  | **INGESTION** | | | **DERMAL** | | | | |
| --- | --- | --- | --- | --- | --- | --- | --- | --- | --- |
| **SP** |  | **ADULT** | | **CHILDREN** | | **ADULT** | | **CHILDREN** | |
|  | **CON** | **CDI** | **HQ** | **CDI** | **HQ** | **CDI** | **HQ** | **CDI** | **HQ** |
| 1 | 0.003 | $8.22\boldsymbol{\times}{10}^{-5}$ | 0.002 | $1.92\boldsymbol{\times}{10}^{-4}$ | 0.005 | $5.92\boldsymbol{\times}{10}^{-5}$ | 0.002 | $1.07\boldsymbol{\times}{10}^{-4}$ | 0.003 |

Supplementary Table 5a: Hazard quotient for adults and children associated with exposure to atrazine concentration in hand dug well water from Oru community

|  | | **INGESTION** | | | | **DERMAL** | | | |
| --- | --- | --- | --- | --- | --- | --- | --- | --- | --- |
|  |  | **ADULT** | | **CHILDREN** | | **ADULT** |  | **CHILDREN** | |
| **SP** | **CON** | **CDI** | **HQ** | **CDI** | **HQ** | **CDI** | **HQ** | **CDI** | **HQ** |
| 1 | 0.01 | $2.74\times{10}^{-4}$ | 0.008 | $6.39\times{10}^{-4}$ | 0.018 | $1.97\times{10}^{-4}$ | 0.006 | $3.58\times{10}^{-4}$ | 0.010 |
| 2 | 0.01 | $2.74\times{10}^{-4}$ | 0.008 | $6.39\times{10}^{-4}$ | 0.018 | $1.97\times{10}^{-4}$ | 0.006 | $3.58\times{10}^{-4}$ | 0.010 |
| 3 | 0.007 | $1.92\times{10}^{-4}$ | 0.005 | $4.47\times{10}^{-4}$ | 0.013 | $1.38\times{10}^{-4}$ | 0.004 | $2.51\times{10}^{-4}$ | 0.007 |
| 4 | ND | ND | ND | ND | ND | ND | ND | ND | ND |
| 5 | ND | ND | ND | ND | ND | ND | ND | ND | ND |
| 6 | ND | ND | ND | ND | ND | ND | ND | ND | ND |
| 7 | 0.01 | $2.74\times{10}^{-4}$ | 0.008 | $6.39\times{10}^{-4}$ | 0.018 | $1.97\times{10}^{-4}$ | 0.006 | $3.58\times{10}^{-4}$ | 0.010 |
| 8 | 0.004 | $1.09\times{10}^{-4}$ | 0.003 | $2.56\times{10}^{-4}$ | 0.007 | $7.89\times{10}^{-5}$ | 0.002 | $1.43\times{10}^{-4}$ | 0.004 |
| 0 | ND | ND | ND | ND | ND | ND | ND | ND | ND |

Supplementary Table 5b: Hazard quotient for adults and children associated with exposure to atrazine concentration in borehole water from Oru community

|  | | **INGESTION** | | | | **DERMAL** | | | |
| --- | --- | --- | --- | --- | --- | --- | --- | --- | --- |
|  |  | **ADULT** | | **CHILDREN** | | **ADULT** |  | **CHILDREN** | |
| **SP** | **CON** | **CDI** | **HQ** | **CDI** | **HQ** | **CDI** | **HQ** | **CDI** | **HQ** |
| 1 | 0.005 | $1.37\times{10}^{-4}$ | 0.004 | $3.19\times{10}^{-4}$ | 0.009 | $9.86\times{10}^{-5}$ | 0.003 | $1.79\times{10}^{-4}$ | 0.005 |
| 2 | BDL | NIL | NIL | NIL | NIL | NIL | NIL | NIL | NIL |
| 3 | ND | ND | ND | ND | ND | ND | ND | ND | ND |
| 4 | 0.004 | $1.09\times{10}^{-4}$ | 0.003 | $2.56\times{10}^{-5}$ | 0.007 | $7.89\times{10}^{-5}$ | 0.002 | $1.43\times{10}^{-4}$ | 0.004 |
| 5 | ND | ND | ND | ND | ND | ND | ND | ND | ND |
| 6 | ND | ND | ND | ND | ND | ND | ND | ND | ND |
| 7 | 0.003 | $8.22\times{10}^{-5}$ | 0.002 | $1.92\times{10}^{-4}$ | 0.005 | $5.92\times{10}^{-4}$ | 0.002 | $1.07\times{10}^{-4}$ | 0.003 |

Supplementary Table 5c: Hazard quotient for adults and children associated with exposure to atrazine concentration in stream water from Oru community

|  | | **INGESTION** | | | | **DERMAL** | | | |
| --- | --- | --- | --- | --- | --- | --- | --- | --- | --- |
|  |  | **ADULT** | | **CHILDREN** | | **ADULT** |  | **CHILDREN** | |
| **SP** | **CON** | **CDI** | **HQ** | **CDI** | **HQ** | **CDI** | **HQ** | **CDI** | **HQ** |
| 1 | 0.004 | $1.09\times{10}^{-4}$ | 0.003 | $2.56\times{10}^{-4}$ | 0.007 | $7.89\times{10}^{-5}$ | 0.002 | $1.43\times{10}^{-4}$ | 0.004 |

**Supplementary Figure 1**; Hazard index for adults and children associated with exposure to atrazine concentrations in hand-dug well, borehole and stream water from Oru community. SP-Sampling point; nd- non-detected

Supplementary Table 6a: Hazard quotient for adults and children associated with exposure to atrazine concentration in hand dug well water from Awa community

|  | | **INGESTION** | | | | **DERMAL** | | | |
| --- | --- | --- | --- | --- | --- | --- | --- | --- | --- |
|  |  | **ADULT** | | **CHILDREN** | | **ADULT** |  | **CHILDREN** | |
| **SP** | **CON** | **CDI** | **HQ** | **CDI** | **HQ** | **CDI** | **HQ** | **CDI** | **HQ** |
| 1 | 0.04 | 1.095 x10^-3^ | 0.003 | 2.6 x10^-3^ | 0.074 | 7.89 x10^-4^ | 0.023 | 1.43 x10^-3^ | 0.040 |
| 2 | 0.02 | 5.48 x10^-4^ | 0.016 | 1.3 x10^-3^ | 0.037 | 3.94 x10^-4^ | 0.011 | 7.15 x10^-3^ | 0.020 |
| 3 | 0.04 | 1.095 x10^-3^ | 0.003 | 2.6 x10^-3^ | 0.074 | 7.89 x10^-4^ | 0.023 | 1.43 x10^-3^ | 0.040 |
| 4 | 0.03 | 8.2 x10^-4^ | 0.003 | 1.9 x10^-3^ | 0.054 | 5.91 x10^-4^ | 0.017 | 1.07 x10^-3^ | 0.030 |
| 5 | 0.04 | 1.095 x10^-3^ | 0.003 | 2.6 x10^-3^ | 0.074 | 7.89 x10^-4^ | 0.023 | 1.43 x10^-3^ | 0.040 |
| 6 | ND | ND | ND | ND | ND | ND | ND | ND | ND |
| 7 | 0.03 | 8.2 x10^-4^ | 0.003 | 1.9 x10^-3^ | 0.054 | 5.91 x10^-4^ | 0.017 | 1.07 x10^-3^ | 0.030 |

Supplementary Table 6b: Hazard quotient for adults and children associated with exposure to atrazine concentration in borehole water from Awa community

|  | | **INGESTION** | | | | **DERMAL** | | | |
| --- | --- | --- | --- | --- | --- | --- | --- | --- | --- |
|  |  | **ADULT** | | **CHILDREN** | | **ADULT** |  | **CHILDREN** | |
| **SP** | **CON** | **CDI** | **HQ** | **CDI** | **HQ** | **CDI** | **HQ** | **CDI** | **HQ** |
| 1 | 0.01 | 2.73 x10^-4^ | 0.0078 | 1.47 x10^-4^ | 0.0042 | 1.97 x10^-4^ | 0.0056 | 3.58 x10^-4^ | 0.0102 |
| 2 | BDL | NIL | NIL | NIL | NIL | NIL | NIL | NIL | NIL |
| 3 | 0.01 | 2.73 x10^-4^ | 0.0078 | 1.47 x10^-4^ | 0.0042 | 1.97 x10^-4^ | 0.0056 | 3.58 x10^-4^ | 0.0102 |
| 4 | 0.01 | 2.73 x10^-4^ | 0.0078 | 1.47 x10^-4^ | 0.0042 | 1.97 x10^-4^ | 0.0056 | 3.58 x10^-4^ | 0.0102 |
| 5 | BDL | NIL | NIL | NIL | NIL | NIL | NIL | NIL | NIL |

**Supplementary Figure 2**; Hazard index for adults and children associated with exposure to atrazine concentrations in hand-dug well, borehole and stream water from Awa community. SP-Sampling point; nd- non-detected

Supplementary Table 7a: Hazard quotient for adults and children associated with exposure to atrazine concentration in hand dug well water from Mamu community

|  | | **INGESTION** | | | | **DERMAL** | | | |
| --- | --- | --- | --- | --- | --- | --- | --- | --- | --- |
|  |  | **ADULT** | | **CHILDREN** | | **ADULT** |  | **CHILDREN** | |
| **SP** | **CON** | **CDI** | **HQ** | **CDI** | **HQ** | **CDI** | **HQ** | **CDI** | **HQ** |
| 1 | 0.04 | $1.09\boldsymbol{\times}{10}^{-3}$ | 0.031 | $2.56\boldsymbol{\times}{10}^{-3}$ | 0.073 | $7.89\boldsymbol{\times}{10}^{-4}$ | 0.023 | $1.43\boldsymbol{\times}{10}^{-3}$ | 0.041 |
| 2 | 0.03 | $8.22\boldsymbol{\times}{10}^{-4}$ | 0.023 | $1.92\boldsymbol{\times}{10}^{-3}$ | 0.055 | $5.92\boldsymbol{\times}{10}^{-4}$ | 0.017 | $1.07\boldsymbol{\times}{10}^{-3}$ | 0.031 |
| 3 | 0.01 | $2.74\boldsymbol{\times}{10}^{-4}$ | 0.008 | $6.39\boldsymbol{\times}{10}^{-4}$ | 0.018 | $1.97\boldsymbol{\times}{10}^{-4}$ | 0.006 | $3.58\boldsymbol{\times}{10}^{-4}$ | 0.010 |

Supplementary Table 7b: Hazard quotient for adults and children associated with exposure to atrazine concentration in borehole water from Mamu community

|  | | **INGESTION** | | | | **DERMAL** | | | |
| --- | --- | --- | --- | --- | --- | --- | --- | --- | --- |
|  |  | **ADULT** | | **CHILDREN** | | **ADULT** |  | **CHILDREN** | |
| **SP** | **CON** | **CDI** | **HQ** | **CDI** | **HQ** | **CDI** | **HQ** | **CDI** | **HQ** |
| 1 | 0.003 | $8.22\boldsymbol{\times}{10}^{-5}$ | 0.002 | $1.92\boldsymbol{\times}{10}^{-4}$ | 0.005 | $5.92\boldsymbol{\times}{10}^{-5}$ | 0.002 | $1.07\boldsymbol{\times}{10}^{-4}$ | 0.003 |

Supplementary Table 7b: Hazard quotient for adults and children associated with exposure to atrazine concentration in stream water from Mamu community

|  | | **INGESTION** | | | | **DERMAL** | | | |
| --- | --- | --- | --- | --- | --- | --- | --- | --- | --- |
|  |  | **ADULT** | | **CHILDREN** | | **ADULT** |  | **CHILDREN** | |
| **SP** | **CON** | **CDI** | **HQ** | **CDI** | **HQ** | **CDI** | **HQ** | **CDI** | **HQ** |
| 1 | BDL | NIL | NIL | NIL | NIL | NIL | NIL | NIL | NIL |
| 2 | 0.003 | $8.22\boldsymbol{\times}{10}^{-5}$ | 0.002 | $1.92\boldsymbol{\times}{10}^{-4}$ | 0.005 | $5.92\boldsymbol{\times}{10}^{-5}$ | 0.002 | $1.07\boldsymbol{\times}{10}^{-4}$ | 0.003 |

**Supplementary Figure 3**; Hazard index for adults and children associated with exposure to atrazine concentrations in hand-dug well, borehole and stream water from Mamu community. SP-Sampling point; nd- non-detected

Supplementary Table 8: Hazard quotient for adults and children associated with exposure to atrazine concentration in hand dug well water from Ilaporu community

|  | | **INGESTION** | | | | **DERMAL** | | | |
| --- | --- | --- | --- | --- | --- | --- | --- | --- | --- |
|  |  | **ADULT** | | **CHILDREN** | | **ADULT** |  | **CHILDREN** | |
| **SP** | **CONC** | **CDI** | **HQ** | **CDI** | **HQ** | **CDI** | **HQ** | **CDI** | **HQ** |
| 1 | 0.01 | $5.48\boldsymbol{\times}{10}^{-4}$ | 0.015 | $6.39\boldsymbol{\times}{10}^{-4}$ | 0.018 | $1.97\boldsymbol{\times}{10}^{-4}$ | 0.006 | $3.58\boldsymbol{\times}{10}^{-4}$ | 0.010 |
| 2 | ND | $\mathrm{ND}$ | ND | $ND$ | ND | $ND$ | ND | $ND$ | ND |
| 3 | 0.003 | $8.22\boldsymbol{\times}{10}^{-5}$ | 0.002 | $1.92\boldsymbol{\times}{10}^{-4}$ | 0.005 | $5.92\boldsymbol{\times}{10}^{-5}$ | 0.002 | $1.07\boldsymbol{\times}{10}^{-4}$ | 0.003 |
| 4 | BDL | NIL | NIL | NIL | NIL | NIL | NIL | NIL | NIL |
| 5 | ND | ND | ND | ND | ND | ND | ND | ND | ND |
| 6 | 0.01 | $5.48\boldsymbol{\times}{10}^{-4}$ | 0.015 | $6.39\boldsymbol{\times}{10}^{-4}$ | 0.018 | $1.97\boldsymbol{\times}{10}^{-4}$ | 0.006 | $3.58\boldsymbol{\times}{10}^{-4}$ | 0.010 |
| 7 | 0.003 | $8.22\boldsymbol{\times}{10}^{-5}$ | 0.002 | $1.92\boldsymbol{\times}{10}^{-4}$ | 0.005 | $5.92\boldsymbol{\times}{10}^{-5}$ | 0.002 | $1.07\boldsymbol{\times}{10}^{-4}$ | 0.003 |
| 8 | ND | ND | ND | ND | ND | ND | ND | ND | ND |
| 9 | ND | ND | ND | ND | ND | ND | ND | ND | ND |
| 10 | ND | ND | ND | ND | ND | ND | ND | ND | ND |

**Supplementary Figure 4**; Hazard index for adults and children associated with exposure to atrazine concentrations in hand-dug well, borehole and stream water from Ilaporu community. SP-Sampling point; nd- non-detected
